# Detecting Presymptomatic Infection Is Necessary to Forecast Major Epidemics in the Earliest Stages of Infectious Disease Outbreaks

**DOI:** 10.1371/journal.pcbi.1004836

**Published:** 2016-04-05

**Authors:** Robin N. Thompson, Christopher A. Gilligan, Nik J. Cunniffe

**Affiliations:** Department of Plant Sciences, University of Cambridge, Cambridge, United Kingdom; Ecole Polytechnique Federale de Lausanne, SWITZERLAND

## Abstract

We assess how presymptomatic infection affects predictability of infectious disease epidemics. We focus on whether or not a major outbreak (i.e. an epidemic that will go on to infect a large number of individuals) can be predicted reliably soon after initial cases of disease have appeared within a population. For emerging epidemics, significant time and effort is spent recording symptomatic cases. Scientific attention has often focused on improving statistical methodologies to estimate disease transmission parameters from these data. Here we show that, even if symptomatic cases are recorded perfectly, and disease spread parameters are estimated exactly, it is impossible to estimate the probability of a major outbreak without ambiguity. Our results therefore provide an upper bound on the accuracy of forecasts of major outbreaks that are constructed using data on symptomatic cases alone. Accurate prediction of whether or not an epidemic will occur requires records of symptomatic individuals to be supplemented with data concerning the true infection status of apparently uninfected individuals. To forecast likely future behavior in the earliest stages of an emerging outbreak, it is therefore vital to develop and deploy accurate diagnostic tests that can determine whether asymptomatic individuals are actually uninfected, or instead are infected but just do not yet show detectable symptoms.

## Author Summary

Emerging epidemics pose a significant challenge to human health worldwide. Accurate real-time forecasts of whether or not initial reports will be followed by a major outbreak are necessary for efficient deployment of control. For all infectious diseases, there is a delay between infection and the appearance of symptoms, i.e. an initial period following first infection during which infected individuals remain presymptomatic. We use mathematical modeling to evaluate the effect of presymptomatic infection on predictions of major epidemics. Our results show rigorously, for the first time, that precise estimates of the current number of infected individuals—and consequently the chance of a major outbreak in future—cannot be inferred from data on symptomatic cases alone. This is the case even if the values of epidemiological parameters, such as the average infection and death or recovery rates of individuals in the population, can be estimated accurately. Accurate prediction is in fact impossible without additional data from which the number of currently infected but as yet presymptomatic individuals can be deduced.

## Introduction

A principal challenge in infectious disease epidemiology is quantifying the threat posed by disease early in emerging outbreaks [1,2]. During the earliest stages of infectious disease outbreaks, two main questions are i) will a major epidemic occur, and ii) what will the final size of the outbreak be [3]? Answering the second of these questions is impossible without understanding the answer to the first. We therefore focus on predicting whether or not reports of initial cases will be followed by a major outbreak of disease, in which a large number of individuals become infected [4–7]. Accurate real-time forecasts at the start of emerging outbreaks are essential for efficient deployment of limited resources for control [8,9]. However, the dynamics of infectious disease outbreaks are influenced by the incubation period, within which hosts are infected but do not yet show symptoms [10–12]. We use mathematical modeling to investigate how the consequent ambiguity in the number of hosts that are currently infected confounds prediction in the earliest stages of a potential major outbreak.

The basic reproductive number, *R*_0_, the average number of secondary cases caused by a single infection in a totally susceptible population, justifiably dominates any discussion of infectious disease epidemiology [4,5,13]. If *R*_0_ < 1, there will certainly not be a major outbreak [14]. When *R*_0_ is above this threshold, however, major outbreaks can but do not always occur [15,16]. In large populations, the distribution of epidemic sizes is bimodal when *R*_0_ > 1, and either the disease dies out with very few ever becoming infected, or it becomes widespread [5,7] (S1 Fig). A major outbreak can therefore naturally be defined as one where the disease becomes widespread, i.e. the total number of hosts that ever become infected lies in the part of the distribution of possible final sizes that contains the larger mode. A well-known approximation to the probability of a major outbreak in a large population can be derived from simple stochastic epidemiological models,

$$\text{Prob}\left(\text{Major outbreak}\right)\;\approx \;1-{\left(\frac{1}{R_{0}}\right)}^{I*},$$

where I* is the number that are infected at the time of estimation [6]. This estimate is widespread in the theoretical epidemiology literature particularly in the case where disease first arrives in the system and so I* = 1 [5,15,17–22]. We note that this formula has also been used in the context of the spread of the recent Ebola outbreak to Nigeria, to estimate the chance that a single undetected infected case will spark a major outbreak [23]. More sophisticated approximations to the probability of a major outbreak can be derived for models containing additional epidemiological detail, for example population structure [24,25], more refined models of individuals’ infectious periods [6], and differences in infectivity between individuals [13]. Crucially, however, the approximation above illustrates that estimates of the probability of a major outbreak require knowledge not only of the values of disease transmission parameters, but also of the total number of currently infected hosts, I*. This includes those individuals that have not yet developed symptoms. Modelers have concentrated on developing increasingly elaborate statistical machinery to estimate the parameters that constitute *R*_0_ [26–28]. Other work, most notably back-calculation [29,30], focuses on estimating the number of individuals that are currently infected, accounting for delays before symptoms emerge. What has never been examined, however, is how the lack of knowledge of precisely how many are infected in the early stages of a potential major outbreak affects predictability of whether or not a large epidemic will in fact go on to occur. In practice, epidemic forecasts for specific pathogens are typically conducted via simulation [11,31–36]. Consequently, we conduct a simulation-based study into the impact of presymptomatic infection—which the formula from the theoretical epidemiology literature suggests might disrupt forecasting—on predictions of major epidemics.

### Case study: Ebola virus disease

As an example of a disease for which initial cases are frequently not followed by major outbreaks, and with a significant delay between infection and emergence of symptoms, we consider Ebola virus disease. All five strains of the genus *Ebolavirus* cause severe acute illness, with early non-specific symptoms including asthenia and myalgia typically followed by nausea, vomiting, hemorrhagic symptoms and, in a significant proportion of cases, death [37]. There are reports of cases of Ebola in remote villages in Central and West Africa every few years [38], hypothesized to be initiated by spillover from reservoirs of infection in wild animal populations, with fruit bats most often implicated as the reservoir host [39]. Often there is no sustained human-to-human transmission, and initial cases do not lead to large outbreaks. However, since 1976 there have been twenty-five distinct reports of primary infection in humans, of which sixteen have led to epidemics causing more than twenty deaths. The largest ever Ebola outbreak started in Guinea in December 2013 and subsequently spread to and caused widespread transmission in Liberia and Sierra Leone, with additional cases in Nigeria, Mali, Senegal, Spain, USA, UK and Italy. This epidemic caused more than 11,000 fatalities before it was declared officially over by the World Health Organization on 14^th^ January 2016, although an additional death was confirmed the following day and additional small flare-ups are still possible [40].

Modeling studies of Ebola have tended to focus on parameter estimation [41–43] and the potential effects of disease control [31,32,37,44]. Here, we instead focus on using an existing epidemiological model fitted to data from the outbreak in Uganda that killed 224 people in 2000 [45] to show how presymptomatic infection affects our ability to predict whether or not reports of initial cases will go on to cause a major outbreak. Ebola is therefore a motivating example for our investigation into how presymptomatic infection affects the predictability of infectious disease epidemics. However, since presymptomatic infection is ubiquitous, our conclusions are applicable to a wide range of pathogens.

## Results

### Estimating the probability of a major outbreak

We use simulations of stochastic compartmental epidemic models to drive our analyses. The models assume that, at any time, every member of the population belongs to a compartment describing their infection and symptom status. In a single realization of the model, whether or not an individual becomes infected is a random process. If an individual does become infected, then the model generates the time at which the individual is first infected, the time at which symptoms first appear and the time at which the individual either dies or recovers. These times are simply those at which the individual passes into the relevant compartments of the model.

**Fig 1:**
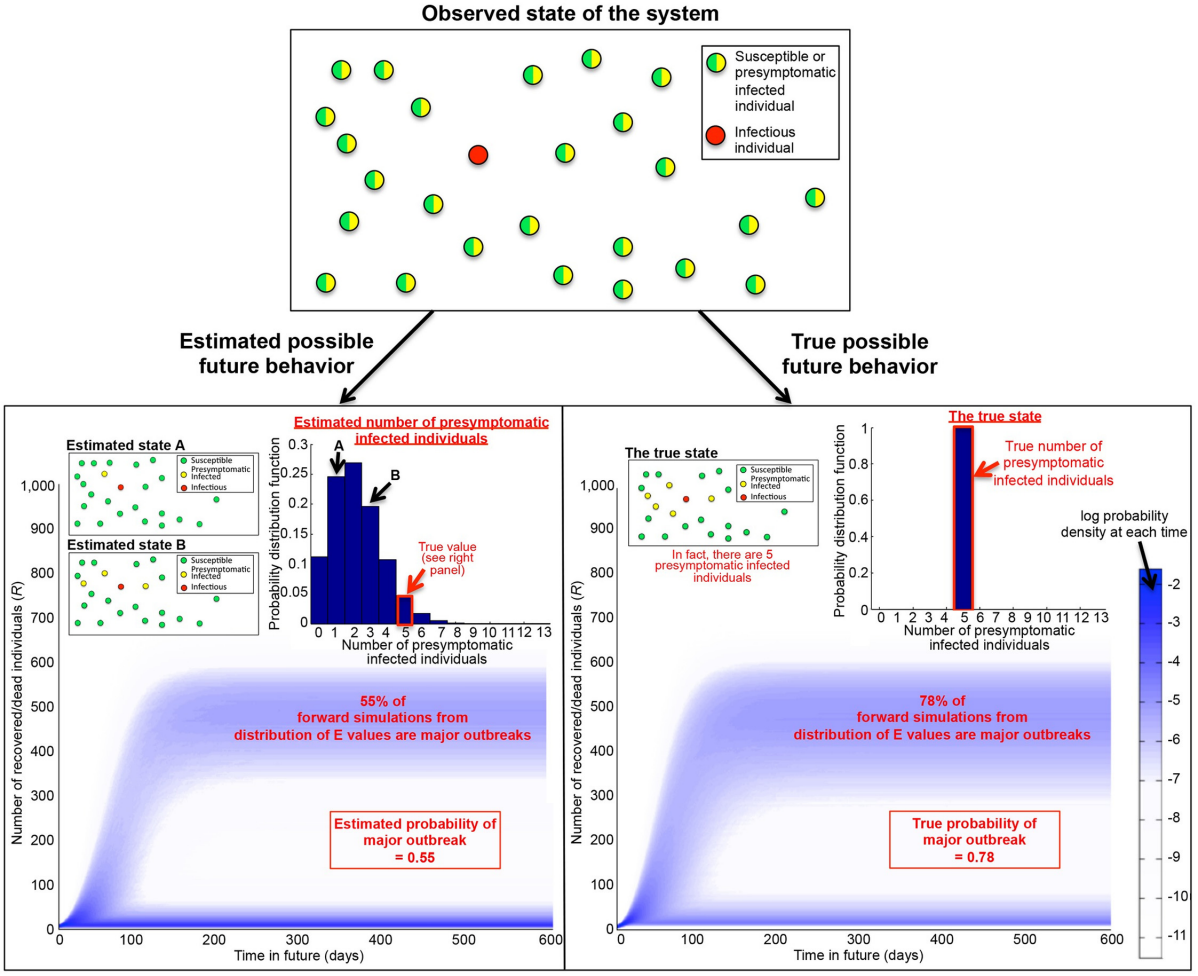
Incorrect estimation of the probability of a major outbreak. The number of presymptomatic infected individuals is estimated (giving a distribution of possible values), and these values are used as initial conditions in forward simulations to build a point estimate of the probability of a major outbreak (bottom left—here, the estimated probability of a major outbreak is 0.55). However, the number of presymptomatic infected individuals actually takes a single value, which can be used in forward simulations to determine the true probability of a major outbreak (bottom right—here, the true probability of a major outbreak is 0.78). The underlying dataset is simulated using the SEIR model, and the predicted future behaviors shown are generated using 100,000 forward simulations of the model.

We therefore produce a “dataset” for the start of an outbreak by running a simulation model. We “freeze” the outbreak at the time of the fourth death, and calculate two quantities using the model (Fig 1): the probability of a major outbreak given complete observation of presymptomatic cases (hereafter referred to as the “true” probability of a major outbreak), and the estimate of this probability that only uses data on the timings of symptoms and deaths and not the times at which individuals are initially infected. In this estimated probability, the presymptomatic cases remain hidden and the number of presymptomatic infected individuals is estimated from the data on symptoms and deaths.

For an individual outbreak, a confidence interval can be constructed around the point estimate that we consider. For the outbreak in Fig 1, the distributional estimate of the number of exposed individuals leads to a 95% confidence interval for the current number of infected individuals of [1,6], which corresponds to an extremely wide 95% confidence interval for the probability of a major outbreak of [0.24,0.78]. The point estimate corresponds to a weighted sum over the distributional estimate of the probability of a major outbreak.

Our initial analysis considers a simplified, SEIR model with exponential waiting times in each compartment. In the SEIR model, presymptomatic infecteds are confined to the uninfectious, latently infected (*E*) class. We relax this assumption later, and also consider the case where the waiting times follow gamma, rather than exponential distributions. Recent modeling work, focused on the recent outbreak of Ebola, often includes considerable epidemiological detail [31,32,44], although there are some exceptions [42,43,46]. Here we take advantage of a previous parameterization of the SEIR model for Ebola, noting that the SEIR model is widely-used for a number of diseases and captures what we want to investigate here—i.e. the effect of presymptomatic infection on major outbreak forecasting—in the simplest possible way.

**Fig 2:**
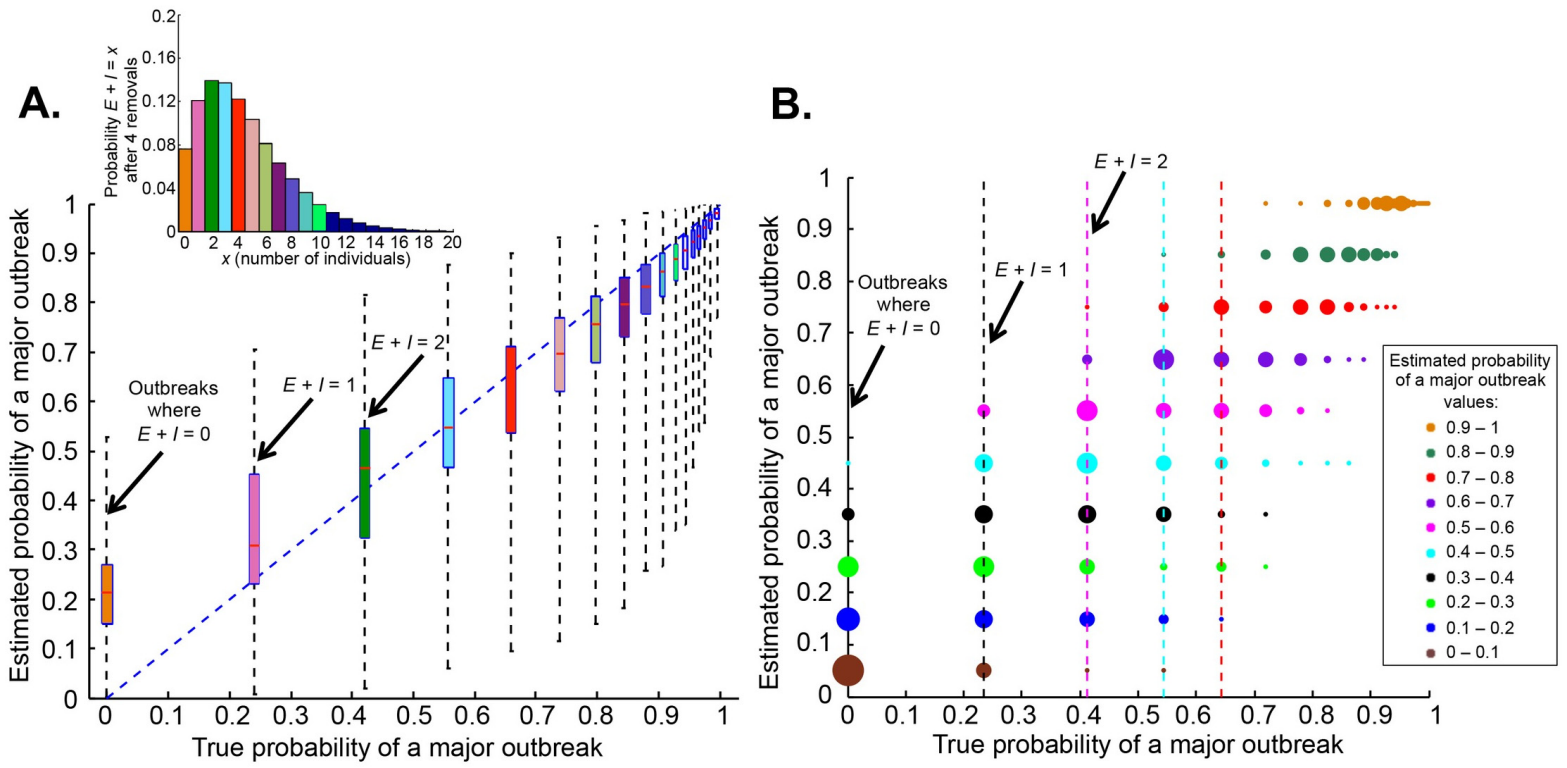
Uncertainty in the probability of a major outbreak when presymptomatic infection cannot be detected. A. Given the true probability of a major outbreak, what point estimate for the estimated probability of a major outbreak might be obtained? (100,000 simulated datasets using the SEIR model for each true probability). For clarity, true probabilities greater than 0.97 are classified into bins of size 0.01. B. How does the true probability of a major outbreak vary between outbreaks with the same point estimate for the probability of a major outbreak? Estimated probabilities are classified into bins of size 0.1 (100,000 simulated datasets per bin). Circle areas are proportional to the number of outbreaks at each true probability, normalized for each bin of estimated probabilities of a major outbreak, so that the sum of the areas of the circles along each horizontal line is constant.

The true and estimated probabilities of a major epidemic are calculated for many simulated outbreaks, to investigate how presymptomatic infection affects the ability to predict major epidemics early in outbreaks (Fig 2A). The probability of a major outbreak depends on the number of infected individuals at the time of estimation (S2 Fig), and hidden presymptomatic infection therefore frustrates prediction. This is even the case when there are actually no presymptomatic infected individuals in the population, since the distribution that estimates the number of presymptomatic individuals will include values other than zero.

In the SEIR model, only discrete values of the true probability of a major outbreak are possible, since the true probability is entirely controlled by the total number of infected individuals at the time of estimation. However, each individual dataset, corresponding to a separate realization of an outbreak, consists of the times that individuals become symptomatic. Slight variations in these times lead to different probability distributions of the number of presymptomatic infecteds. These differences are reflected in the estimated probability of a major outbreak. Consequently, the estimated probability for each true value is effectively a continuously varying quantity.

The key qualitative result, i.e. that the estimated and true probabilities of a major outbreak do not match, is robust to performing estimation at different stages of the start of an outbreak, and to different lengths of the incubation period and values of *R*_0_ (S3–S6 Figs). Additional uncertainty in the probability of a major outbreak occurs when the parameters for disease spread must also be estimated from the transmission data (S7 Fig). However, no matter how much parameter estimation is improved, for example using data from previous outbreaks to inform estimates, presymptomatic infection still causes significant errors in forecasting major outbreaks.

The problem of practical interest for an emerging epidemic is inferring the true probability of a major outbreak. For an individual outbreak, a (often imprecise) confidence interval can be constructed around the point estimate as we described above. However, we characterize the implications of presymptomatic infection more generally by examining many simulated outbreaks, inverting our point estimate of the probability of a major outbreak to consider the range of true probabilities that are possible for each estimated value. Similar estimated probabilities of a major outbreak can correspond to a remarkably wide range of true probabilities (Fig 2B). For example, for outbreaks in which the estimated probability is between 0.5 and 0.6, the true probability can lie between 0.23 and 0.83. We note the extreme values are themselves quite likely: in 13% of these simulated outbreaks, the true probability is in fact either 0.23 or 0.83.

### Improving estimates using diagnostic tests

**Fig 3:**
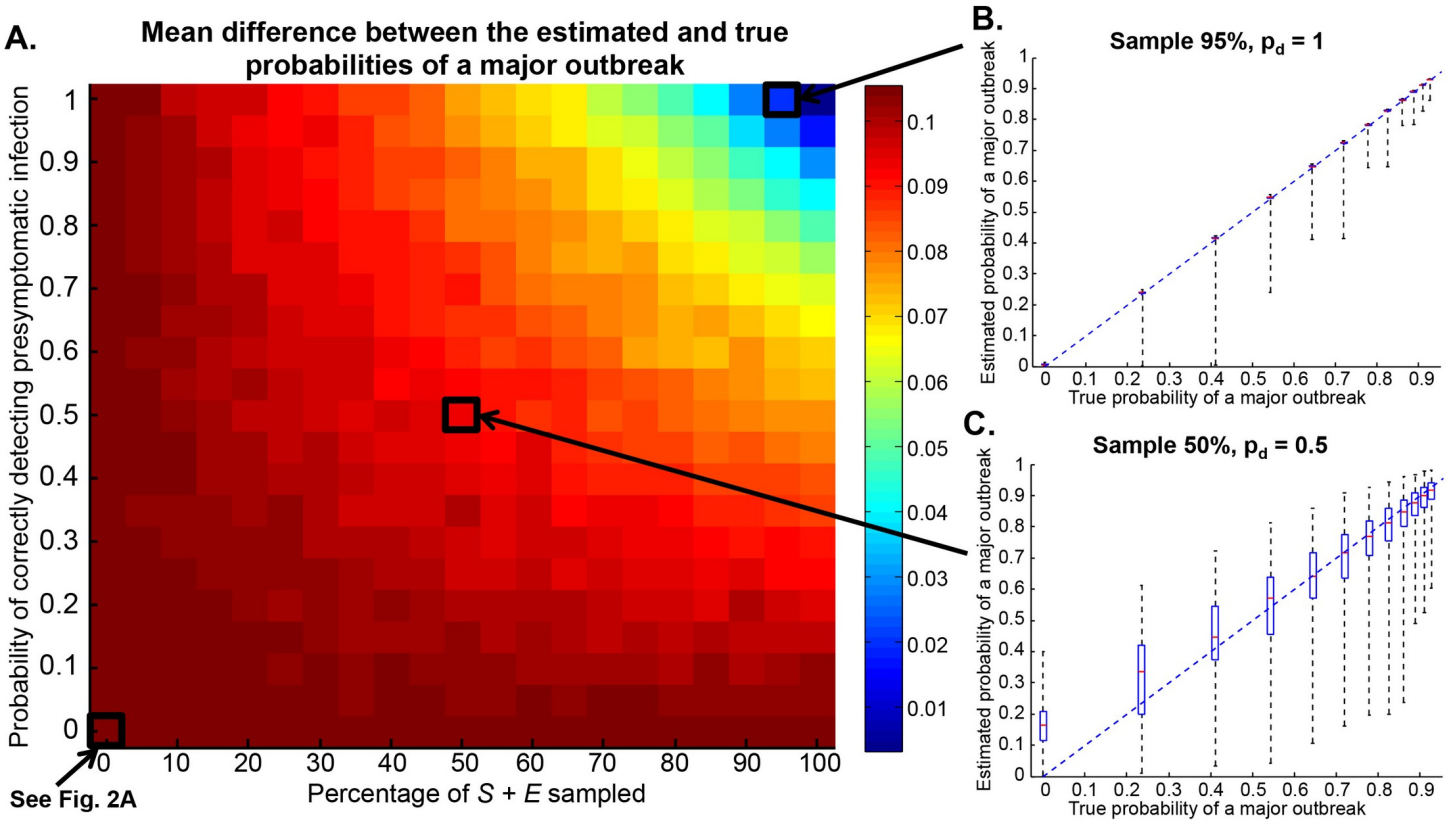
Error in estimating the probability of a major outbreak using the SEIR model when asymptomatic individuals are tested for infection. A. The mean error in the probability of a major outbreak, as a function of the percentage of asymptomatic individuals tested and the probability that presymptomatic infection is correctly identified (calculated from 10,000 simulations for each (percentage, *p*_*d*_) pair). B. Variation in estimates of the probability of a major outbreak when 95% of asymptomatic individuals are tested and the test is perfectly reliable. C. Variation in estimates of the probability of a major outbreak when 50% of asymptomatic individuals are tested and presymptomatic infection is correctly identified 50% of the time.

Estimation of the chance of a major outbreak can be improved by the use of diagnostic tests to determine whether asymptomatic individuals are susceptible or presymptomatic infected. Since the reliability of diagnostic tests affects the extent to which forecasting is improved (Fig 3), it is not only important to develop diagnostic tests but also to ensure their continued refinement. To illustrate the general principle that diagnostic tests could be used to improve prediction, we simply choose individuals to test at random from the asymptomatic individuals in the population. With random selection, the diagnostic test must be deployed widely to reduce the error in estimates significantly, although of course careful choice of which individuals to test (e.g. via contact tracing) would reduce the need for such widespread deployment in practice.

### Different assumptions about the incubation and latent periods

**Fig 4:**
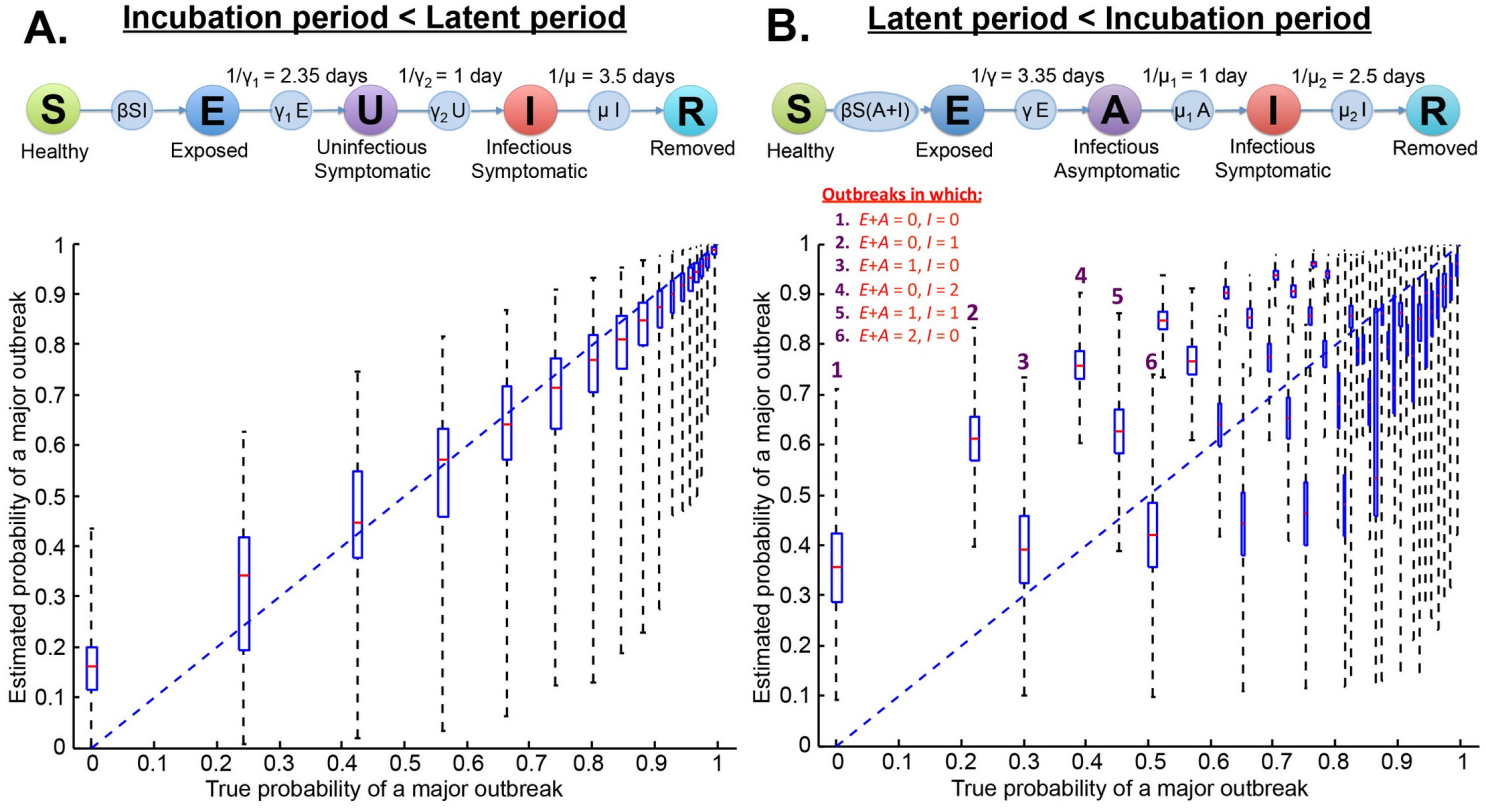
Estimating the probability of a major outbreak when incubation and latent periods are not identical. A. Symptoms occur before infectiousness. B. Symptoms occur after infectiousness. In B, the number of presymptomatic infected individuals is estimated using reversible jump Markov chain Monte Carlo [48]. In the boxplots in A, for clarity, true probabilities greater than 0.98 are classified into bins of size 0.01, and in B, true probabilities greater than 0.8 are classified into bins of size 0.01.

The emergence of symptoms and the emergence of infectivity are assumed to coincide in the SEIR model. We relax this assumption by considering two other models. In the first, individuals display symptoms before becoming infectious (Fig 4A). In the second, individuals are infectious before becoming symptomatic (Fig 4B). When symptoms appear before individuals are infectious, the incubation period is reduced, so more infected individuals can be detected. As a result, predictions of major outbreaks become more accurate, although some systematic ambiguity nevertheless remains (Figs 4A and S8A). Conversely, if the incubation period is instead longer than the latent period, as is the case for many human diseases [47], it becomes more difficult to predict major outbreaks accurately (Figs 4B and S8B).

In Fig 4B, the variable heights of adjacent boxplots indicate that the distribution of infected individuals between the asymptomatic and symptomatic infectious classes affects the estimated probability of a major outbreak. For example, the heights of the second and third boxplots from the left can be explained as follows. Consider two outbreaks, each with only a single infected individual at the time that the chance of a major outbreak is being estimated. Suppose that in the first outbreak (“outbreak one”, say), the infected individual is presymptomatic, but in the second outbreak (“outbreak two”) the infected individual is symptomatic. In outbreak two, because disease is observed since the infected individual is symptomatic, the estimated probability of a major outbreak will be high compared to outbreak one. However, whilst the estimated probability of a major outbreak is higher for outbreak two, the true probability of a major outbreak is in fact higher for outbreak one. This is because, in Fig 4B, individuals can be infectious both when they are presymptomatic and when they are symptomatic. A presymptomatic individual is therefore likely to be infectious for a longer period in future than a symptomatic individual. A longer time infectious corresponds to (on average) more infections, and therefore a higher true probability of a major outbreak.

It might also naïvely be thought that prediction would be easiest in outbreaks in which many infected individuals are symptomatic. However, when a large proportion of infected individuals are symptomatic, the total number of infected individuals tends to be overestimated, causing large errors in forecasts (*cf*. the boxplots corresponding to a single infected individual at the time of estimation in Fig 4B).

The default assumption for compartmental models is that incubation and infectious periods are exponentially distributed. We relax this assumption, and draw periods instead from two-parameter gamma distributions to reflect the observed incubation and infectious periods for a number of diseases [49,50] (S9 Fig). Recently-infected individuals are more likely to remain infectious for a long period beyond the time of estimation than individuals that have already been infected for a long time. Consequently, outbreaks with many presymptomatic infecteds have a high true probability of a major outbreak. However, because presymptomatic individuals are unobserved, the estimated probability of a major outbreak is low in these outbreaks.

## Discussion

Predicting whether or not a major epidemic is likely, from the limited data typically available during the first few days of an outbreak, has received surprisingly little attention. A notable exception is the paper by Drake [51], which shows that the exact final size varies significantly between simulated outbreaks under identical conditions. He investigates how this variability scales with the contact rate between individuals and the efficacy and speed of control responses. However an incubation period is not explicitly included in the model used. Craft *et al*. [7] use a model of rabies in canids to show that the first four death times cannot be used to forecast major outbreaks. However, by assuming that the data consist of death times alone, the factors potentially responsible for this imprecision are confounded. Neither Drake [51] nor Craft *et al*. [7] quantify the error caused by presymptomatic infection. In addition to quantifying this error, our main message is that presymptomatic infection by itself is sufficient to cause error in predictions of whether or not an outbreak will be major, let alone in predicting the final size exactly. This error is particularly notable when there are no infected individuals in the population at all (i.e. the outbreak has already faded out), since the distribution that estimates the number of presymptomatic infected individuals will include values other than zero.

To focus entirely on the uncertainty caused by presymptomatic infection, we worked in an idealized setting in which symptomatic cases and deaths were recorded perfectly and in which the values of disease transmission parameters were known exactly. This allowed us to calculate the exact probability distribution of the current size of the outbreak, i.e. the total number of individuals currently infected, given that presymptomatic infection causes some infected individuals to be unobservable. This distribution drives the estimated probability of a major outbreak.

In practice, however, the distribution of possible current outbreak sizes would have to be estimated from incomplete data on symptomatic cases and deaths, without exact knowledge of parameter values and sometimes without even knowing the total population size precisely. One method for doing this is back-calculation, as originally designed by Brookmeyer and Gail for HIV-AIDS [29], which provides an estimate for the distribution of possible current outbreak sizes. Although, to the best of our knowledge, back-calculation has not been used to estimate the probability of a major outbreak, such a forecast using back-calculation as an input would necessarily be less precise than those used in our analyses here, since we have used the exact distribution of current outbreak sizes given presymptomatic infection. Indeed, by restricting our attention to the case in which there are sufficient data that the number of presymptomatic individuals is the only quantity being estimated, our results provide an upper bound on the ability of any method that seeks to predict major outbreaks from data on symptomatic cases alone. In fact, given the extensive knowledge of the epidemic assumed here, the basic formulation of back-calculation can be extended in a natural fashion to obtain the exact probability distribution of the current size of the outbreak that we use to generate our estimates for the probability of a major outbreak (S4 Text, S10 Fig).

Prediction during the recent Ebola outbreak has been criticized for overestimating the total number of cases that actually occurred [52]. Similarly, modeling studies during the 2009 H1N1 outbreak typically overestimated the total number of cases [53]. In contrast with investigations that attempt to predict the final epidemic size, we differentiated only between “minor” and “major” outbreaks. Our focus was prediction during the very early stages of an outbreak, before a major outbreak is underway, rather than forecasting the final extent of a major outbreak once the epidemic has taken off. This very initial phase of outbreaks is particularly important given the recent interest in rapid detection of disease outbreaks [54–57].

We assumed that the parameter values controlling disease spread are unchanged throughout the early stage of the outbreak, whereas in reality these parameters might vary temporally in response to changing contact networks and control interventions [58], as well as varying environmental conditions [59]. However, any such variations will only exacerbate the uncertainty that we have shown exists. Other sources of uncertainty such as under-reporting, which has posed a challenge to forecasting during the recent Ebola outbreak [60], will also decrease predictability further, although as we have shown presymptomatic infection alone is sufficient to make precise prediction impossible. A systematic investigation of the errors in forecasting caused by under-reporting in comparison to those due to other features such as presymptomatic infection or epidemiological parameter uncertainty is a possibility for a future study.

Our work shows rigorously, for the first time, that no matter how accurately disease transmission parameters are estimated, precise estimates early in outbreaks of whether a major epidemic will occur will remain unavailable without data about presymptomatic infection. This is still the case even if significant resources are devoted to recording symptomatic cases accurately. Consequently, diagnostic tests that can identify presymptomatic infecteds [61,62] are extremely important for improving forecasts of epidemic outbreaks. While our simulations consider random testing of asymptomatic individuals, in practice testing is costly [63], so it is vital that predictability is further improved in a cost-effective way by careful selection of individuals to test. This could be done by contact tracing [64] or using statistical methods to identify individuals with the highest risk of being infected [65], although of course effective and cheap diagnostics are still required. A systematic investigation into which asymptomatic individuals ought to be tested, accounting for the specificity of the tests as well as the sensitivity, would be a valuable extension to our work. A recent analysis of Ebola [66] has considered testing of individuals already exhibiting symptoms to confirm whether the patients have Ebola or a different disease with similar symptoms. That study shows that using rapid diagnostic tests in combination with slower but more accurate diagnostic tests could have significantly reduced the number of cases in Sierra Leone in the recent outbreak.

Our conclusions are robust to various characteristics of the disease, and so apply to all infectious diseases. We chose to use Ebola as a representative case study, but our results are in fact generic. In particular, our key message that presymptomatic infection drives uncertainty in whether an emerging outbreak will become major holds throughout the early stages of the outbreak (S3 and S4 Figs), as well for a number of values of the basic reproduction number of the pathogen (S6 Fig). For Ebola, there is debate as to whether the onset of symptoms and infectiousness coincide [67] or not [68]. However, symptoms and infectiousness are certainly not always concurrent: HIV is a high profile example, for which the time between infection and recognizable symptoms can take years [69], whereas individuals are infectious within months of acquiring the virus [70]. We have considered different models in which symptoms and infectiousness are not assumed to coincide (Figs 4 and S8). While we showed prediction is most reliable for diseases for which the incubation period is shorter than the latent period, even very short incubation periods can generate significant uncertainty in the number of presymptomatic infecteds, and therefore the probability of a major outbreak (S5 Fig). This means that our conclusions even hold for diseases such as influenza and norovirus, which have incubation periods of only a few days [4]. The messages we have set out are also robust to different distributions of the incubation and infectious periods, as we showed by considering models for which these periods follow gamma rather than exponential distributions (S9 Fig).

Of course, our conclusions are relevant to pathogens of agricultural and wild animals and plants, as well as humans. *Xylella fastidiosa* is a plant pathogen that is currently invading southern Italy, causing devastating damage to olive groves [71]. Containment and surveillance zones have been set up in an attempt to find the pathogen and subsequently mitigate spread via control interventions. Surveys in the containment zone do include some laboratory testing for presymptomatic infection, with the surveillance zone solely relying on diagnosis from visual inspection [72]. We have shown that consideration of presymptomatic infection is extremely important when forecasting the spread of pathogens, and so it is also likely to be important when planning interventions that attempt to slow or prevent spread. Studies examining the impacts of presymptomatic infection on forecasting and control of specific pathogens would represent valuable applied extensions to this publication.

At the time of writing, a point-of-care diagnostic test that can detect Ebola from blood samples has been developed and found to be accurate [73]. In light of our analysis, the continued development, deployment and improvement of this and other diagnostic tests that determine whether asymptomatic individuals are infected is of obvious public health importance, not only for Ebola but also for other infectious diseases.

## Methods

### Mathematical models

We perform our analyses using stochastic compartmental models of disease spreading in a small population. Here we outline the three types of model we use: the standard SEIR model, which assumes that symptoms and infectiousness coincide; more complex models that relax this assumption; and a model that assumes that the incubation and infectious periods follow gamma, rather than exponential, distributions.

#### Equal incubation and latent periods (SEIR model)

For simplicity we use a SEIR model initially, making the commonly-used assumption that the emergence of symptoms and of infectivity coincide exactly [41,45,74]. The classic deterministic SEIR model has the following form [5]:

$$\begin{array}{l} \frac{\text{d}S}{\text{d}t}=-\beta SI,\\ \frac{\text{d}E}{\text{d}t}=\beta SI-\gamma E,\\ \frac{\text{d}I}{\text{d}t}=\gamma E-\mu I,\\ \frac{\text{d}R}{\text{d}t}=\mu I. \end{array}$$

Here, *S* is the number of individuals susceptible to the pathogen, *E* the number latently and presymptomatically infected, *I* the number of symptomatic infectious individuals, and *R* the number of dead or recovered individuals. We conduct our analysis starting from one initial presymptomatic infected (with the rest of the population of size 1,000 susceptible), using the analogous stochastic model, and generate simulations using the Gillespie direct method [75]. The model is parameterized for Ebola [45]: *β* = 3.83 × 10^−4^ days^−1^, 1/*γ* = 3.35 days and 1/*μ* = 3.5 days (so that *R*_0_ ≈ 1.34). However, we also test the robustness of our results to these choices. The World Health Organization states that the incubation period is usually between 2 and 21 days [76]. The mean value from the fitted model we have used is at the lower end of this interval and therefore provides a particularly stringent test of the possible effects of presymptomatic infection on forecasting major outbreaks of disease.

#### Unequal incubation and latent periods (SEUIR and SEAIR models)

The SEIR model assumes that the incubation and latent periods coincide exactly, so that individuals in the *I* class are both infectious and symptomatic. For Ebola, there is debate as to whether the onset of symptoms and infectiousness coincide [67] or not [68]; however models usually assume that they do [32,37,44]. This assumption is certainly untrue for numerous other diseases [4]. We therefore also consider models in which the incubation and latent periods are unequal, thereby allowing for asymptomatic infectious or symptomatic uninfectious individuals. To examine the case where symptoms appear before infectiousness, we use the stochastic SEUIR model, in which individuals in the *U* (i.e. Uninfectious symptomatic) class are symptomatic but not yet infectious. To illustrate the effects of this extra compartment, we make the representative choice that the average time spent in the *E* class in the SEIR model case is now split between 1/*γ*_*1*_ = 2.35 days in the *E* class and 1/*γ*_*2*_ = 1 day in the *U* class. To consider potential effects of individuals becoming infectious before symptoms appear, we use the stochastic SEAIR model, in which individuals in the *A* (i.e. Asymptomatic infectious) class are asymptomatic but infectious. In this case, we assume that the average time spent in the equivalent of the *I* class in the SEIR model is now split between 1/*μ*_*1*_ = 1 day in the *A* class and 1/*μ*_*2*_ = 2.5 days in the *I* class.

#### Gamma-distributed incubation and infectious periods

We consider a model that uses the so-called “method of stages” [6,77] to replace exponential distributions for the incubation and infectious periods with gamma distributions, by replacing the *E* and *I* classes in the SEIR model with three *E* and *I* classes (each with period one third of the respective original class). Gamma distributions are more realistic for a number of infectious diseases [49,50], and have been found to fit incubation period and time from symptom onset to death data from the recent Ebola outbreak [67].

### Estimating the number of presymptomatic infected individuals

Since our concern is quantifying uncertainty caused by presymptomatic infection alone, we assume that the parameters controlling disease transmission are known, and that complete data are available from the very beginning of the epidemic for changes in the number of symptomatic infected individuals over time. These data can be used to construct the probability distribution for the number of presymptomatic infected individuals at the time of estimation (S1 Text). For the SEIR model, the data on symptomatic cases are used to estimate the probability that an asymptomatic individual is infected, which feeds into a binomial distribution to estimate the number of presymptomatic infected individuals. The approach can readily be adapted for the SEUIR and gamma-distributed incubation and infectious periods cases. In the SEAIR model case, the *A* class causes the complete time series of infectious individuals to be unobserved, so that the required probability cannot be calculated. Instead reversible jump Markov chain Monte Carlo (S2 Text) is used to estimate the probability distribution for the number of currently infected individuals.

### Testing to detect presymptomatic infection

To illustrate the principle that diagnostic tests can improve forecasts, the sampling of asymptomatic individuals and testing to find presymptomatic infection is modeled by choosing individuals at random out of the *S* or *E* classes without replacement. If the individual is susceptible, then infection is not detected (i.e. the test produces no false positives), whereas if the individual is presymptomatic infected, the pathogen is detected with probability *p*_*d*_. The results of the sample can then be integrated into the estimate of the probability distribution of the number of presymptomatic infected individuals, which therefore becomes more precise (S3 Text).

### The true and estimated probability of a major outbreak

We estimate two probabilities using data from individual simulated epidemics at the time of the fourth death: the true probability of a major outbreak, and the best point estimate of this probability consistent with the transmission data. Specifically, we calculate the true probability of an outbreak by “freezing” the infection status of all individuals at the time of four deaths, simulating a very large number of outbreaks (100,000) using these data as initial conditions, and finding the proportion of simulations in which a major outbreak occurs (defined as more than 10% of the population ever becoming infected, *cf*. S1 Fig). Of course, this calculation is only possible since the number of presymptomatic infected individuals is known.

To calculate the estimated probability of a major outbreak, we instead imagine that the exact infection statuses of individuals that are asymptomatic (i.e. susceptible individuals and presymptomatic infected individuals) are unknown, as would be the case in practice. We use the data on symptomatic cases up to the time of the fourth death to infer the probability distribution of the number of presymptomatic infecteds. We then calculate the estimated probability of a major outbreak by running an ensemble of simulations that sample initial conditions from this distribution on each forward run.

## Supporting Information

S1 Fig Possible outbreak sizes for different values of *R*_0_ in the stochastic SEIR model. Probability distributions for the total number of individuals ever infected for various values of *R*_0_, obtained from 100,000 simulated outbreaks per *R*_0_ value (starting with one presymptomatic infected individual and all other individuals in the population of size 1,000 susceptible). Other parameter values: 1/*γ* = 3.35 days and 1/*μ* = 3.5 days.
(PDF)

S2 Fig Estimating the probability of a major outbreak when the total number of infected individuals is known (i.e. the number of presymptomatic infecteds is known exactly). When the total number of infected individuals is known, the probability of a major outbreak can be estimated accurately. The boxplots reduce to a horizontal line for each true probability of a major outbreak. Data obtained from 100,000 simulated SEIR model datasets for each true probability of a major outbreak; this is the case for all Supporting Information figures containing box plots.
(PDF)

S3 Fig Robustness of results to different numbers of deaths at the time of estimation. For clarity, true probabilities greater than 0.97 are classified into bins of size 0.01.
(PDF)

S4 Fig Evolution of error in estimates of the probability of a major outbreak as the outbreak persists. A. Error in estimates of the probability of a major outbreak as a function of the age of the outbreak (*x* deaths). Inset: Probability of an individual outbreak persisting until at least *x* deaths. B. Average number of *E* and *I* after *x* deaths in simulated outbreaks in which at least *x* deaths occur. Each boxplot in A and bar in B is obtained from 100,000 simulations of the stochastic SEIR model in which at least *x* deaths occur. Each bar in the inset to A is obtained from 100,000 simulations of the stochastic SEIR model.
(PDF)

S5 Fig Robustness of results to different ratios of incubation to infectious period. For extremely short incubation periods, the probability of a major outbreak can be estimated more accurately (since variation in the number of presymptomatic infected individuals between simulations is lower). Here, the infectious period is held fixed and the incubation period varied so that the ratio of these is consistent with poliomyelitis (ratio = 0.12), influenza (ratio = 0.8), mumps (ratio = 2.5) and diphtheria (ratio = 5) [4]. True probabilities greater than 0.97 are classified into bins of size 0.01.
(PDF)

S6 Fig Robustness of results to different values of the basic reproductive number. *R*_0_ is varied by changing the infection rate, *β*, between subfigures. For *R*_0_ = 1.2, true probabilities greater than 0.97 are classified into bins of size 0.01. For *R*_0_ = 1.6, true probabilities greater than 0.98 are classified into bins of size 0.01. For *R*_0_ = 2 and *R*_0_ = 4, true probabilities greater than 0.99 are classified into a bin of size 0.01.
(PDF)

S7 Fig Error in the probability of a major outbreak when the infection rate is estimated. If the probability of a major outbreak is estimated with *β* unknown but the number of presymptomatic infected individuals at the time of estimation known, then there can be less error than due to presymptomatic infection alone. Here, *β* is estimated via maximum likelihood estimation, and constrained to within *x*% of the true value (representing prior knowledge about the value of *β*). Constructed from 10,000 outbreaks simulated until four deaths have occurred using the SEIR model.
(PDF)

S8 Fig Error in estimating the probability of a major outbreak when asymptomatic individuals are tested for infection, when the incubation and latent periods are not identical. The mean error in the probability of a major outbreak, as a function of the percentage of asymptomatic individuals tested and the probability that presymptomatic infection is correctly identified, is calculated from 10,000 simulations for each (percentage, *p*_*d*_) pair when: A. Symptoms appear before individuals become infectious; B. Symptoms appear after individuals become infectious.
(PDF)

S9 Fig Estimating the probability of a major outbreak with gamma distributed incubation and infectious periods. The incubation and infectious periods are both split into three classes, each with exponentially distributed waiting times. Since the true probability of a major outbreak is no longer restricted to discrete values, we classify the true probabilities into bins. The bins are of size 0.1 for true probabilities greater than 0.3. Since lower true probabilities occur infrequently, for computational efficiency we then consider true probabilities equal to 0, and true probabilities greater than zero but less than 0.3, in their own bins.
(PDF)

S10 Fig Using a single simulated dataset to compare the exact probability distribution for the current epidemic size given idealized data, as used in our paper, with approximations of this distribution obtained using back-calculation (see S4 Text). A. The dataset used for estimating *E* (the true value of *E* at the time of estimation is 2). B. Estimation of *E* using simple back-calculation. As can be seen, simple back-calculation does not discriminate well between different small values of *E*, which is important in estimating the probability of a major outbreak when the first few symptomatic cases occur. C. Estimation of *E* using extended back-calculation (with the full *I(t)* curve observed, and population size and transmission parameters known). Extended back-calculation captures the exact distribution used in our manuscript.
(PDF)

S1 Text Estimating the number of presymptomatic infected individuals. (PDF)

S2 Text Estimating the number of individuals in each compartment of the SEAIR model. (PDF)

S3 Text Improving estimates by sampling to find presymptomatic infection. (PDF)

S4 Text Consistency of our estimates with an extended version of back-calculation. (PDF)
