# Supplementary material for: Detecting Presymptomatic Infection Is Necessary to Forecast Major Epidemics in the Earliest Stages of Infectious Disease Outbreaks
Source: PLoS Comput Biol. 2016 Apr 5;12(4):e1004836. doi: 10.1371/journal.pcbi.1004836 (PMC4821482; doi:10.1371/journal.pcbi.1004836)

S1 Fig. Possible outbreak sizes for different values of R_0 in the stochastic SEIR model. Probability distributions for the total number of individuals ever infected for various values of R_0, obtained from 100,000 simulated outbreaks per R_0 value (starting with one presymptomatic infected individual and all other individuals in the population of size 1,000 susceptible). Other parameter values: $1/\gamma$ = 3.35 days and $1/\mu$ = 3.5 days.

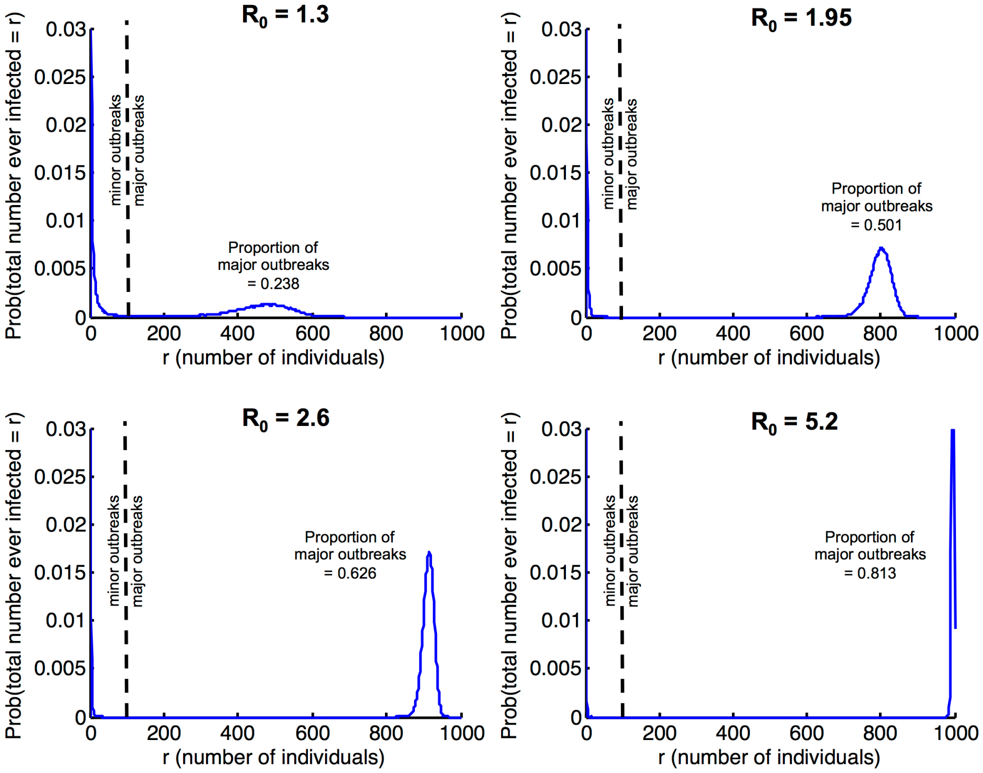

Supplement: S1 Fig — Probability distributions for the total number of individuals ever infected for various values of R0, obtained from 100,000 simulated outbreaks per R0 value (starting with one presymptomatic infected individual and all other individuals in the population of size 1,000 susceptible). Other parameter values: 1/γ = 3.35 days and 1/μ = 3.5 days. (PDF) [file pcbi.1004836.s001.pdf]
