# Supplementary material for: Detecting Presymptomatic Infection Is Necessary to Forecast Major Epidemics in the Earliest Stages of Infectious Disease Outbreaks
Source: PLoS Comput Biol. 2016 Apr 5;12(4):e1004836. doi: 10.1371/journal.pcbi.1004836 (PMC4821482; doi:10.1371/journal.pcbi.1004836)

S2 Fig. Estimating the probability of a major outbreak when the total number of infected individuals is known (i.e. the number of presymptomatic infecteds is known exactly). When the total number of infected individuals is known, the probability of a major outbreak can be estimated accurately. The boxplots reduce to a horizontal line for each true probability of a major outbreak. Data obtained from 100,000 simulated SEIR model datasets for each true probability of a major outbreak; this is the case for all Supporting Information figures containing box plots.

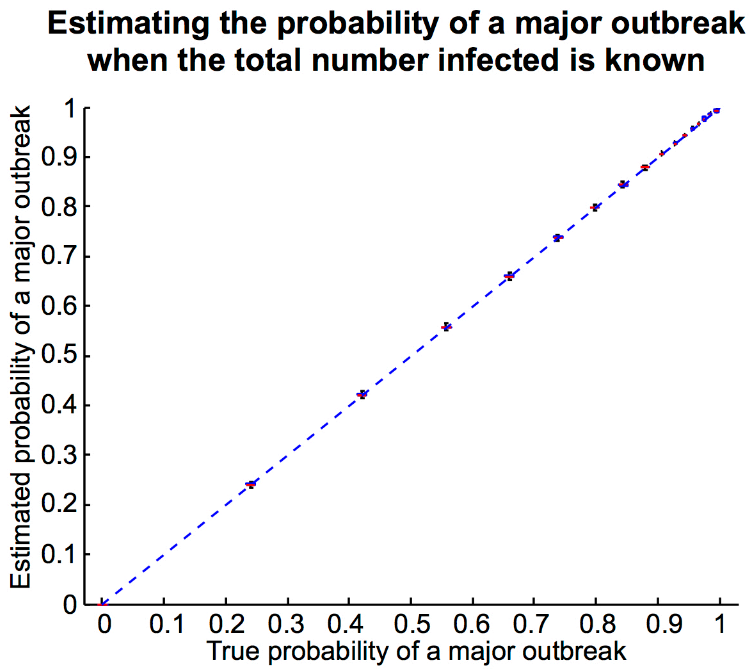

Supplement: S2 Fig — When the total number of infected individuals is known, the probability of a major outbreak can be estimated accurately. The boxplots reduce to a horizontal line for each true probability of a major outbreak. Data obtained from 100,000 simulated SEIR model datasets for each true probability of a major outbreak; this is the case for all Supporting Information figures containing box plots. (PDF) [file pcbi.1004836.s002.pdf]
