# Supplementary material for: Detecting Presymptomatic Infection Is Necessary to Forecast Major Epidemics in the Earliest Stages of Infectious Disease Outbreaks
Source: PLoS Comput Biol. 2016 Apr 5;12(4):e1004836. doi: 10.1371/journal.pcbi.1004836 (PMC4821482; doi:10.1371/journal.pcbi.1004836)

S3 Fig. Robustness of results to different numbers of deaths at the time of estimation. For clarity, true probabilities greater than 0.97 are classified into bins of size 0.01.

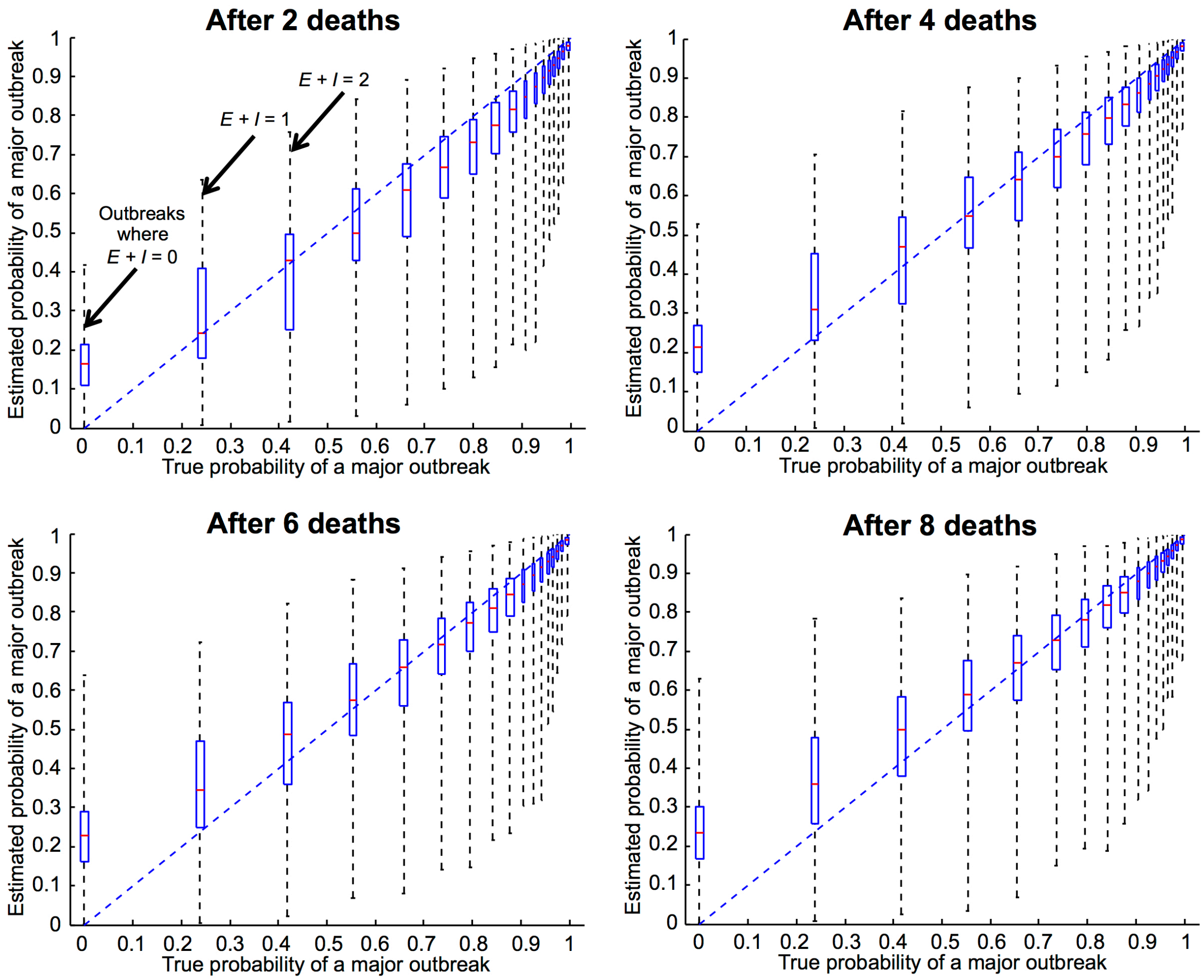

Supplement: S3 Fig — For clarity, true probabilities greater than 0.97 are classified into bins of size 0.01. (PDF) [file pcbi.1004836.s003.pdf]
