# Supplementary material for: Detecting Presymptomatic Infection Is Necessary to Forecast Major Epidemics in the Earliest Stages of Infectious Disease Outbreaks
Source: PLoS Comput Biol. 2016 Apr 5;12(4):e1004836. doi: 10.1371/journal.pcbi.1004836 (PMC4821482; doi:10.1371/journal.pcbi.1004836)

S4 Fig. Evolution of error in estimates of the probability of a major outbreak as the outbreak persists. A. Error in estimates of the probability of a major outbreak as a function of the age of the outbreak (*x* deaths). Inset: Probability of an individual outbreak persisting until at least *x* deaths. B. Average number of *E* and *I* after *x* deaths in simulated outbreaks in which at least *x* deaths occur. Each boxplot in A and bar in B is obtained from 100,000 simulations of the stochastic SEIR model in which at least *x* deaths occur. Each bar in the inset to A is obtained from 100,000 simulations of the stochastic SEIR model.

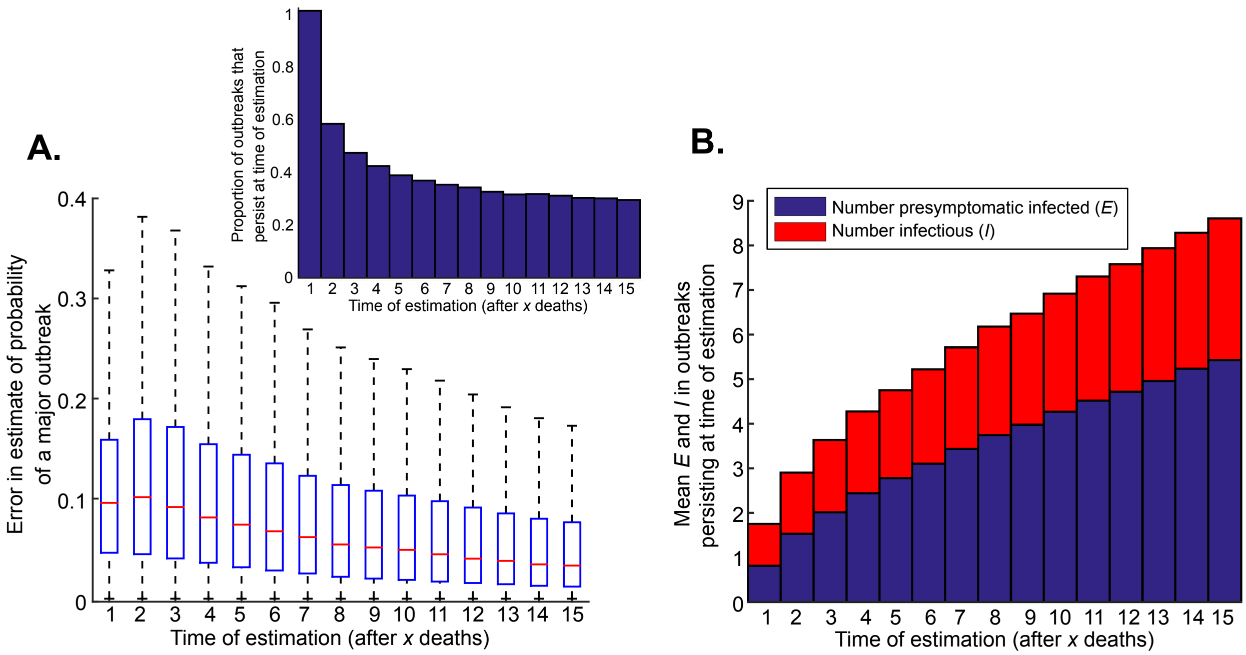

Supplement: S4 Fig — A. Error in estimates of the probability of a major outbreak as a function of the age of the outbreak (x deaths). Inset: Probability of an individual outbreak persisting until at least x deaths. B. Average number of E and I after x deaths in simulated outbreaks in which at least x deaths occur. Each boxplot in A and bar in B is obtained from 100,000 simulations of the stochastic SEIR model in which at least x deaths occur. Each bar in the inset to A is obtained from 100,000 simulations of the stochastic SEIR model. (PDF) [file pcbi.1004836.s004.pdf]
