# Supplementary material for: Detecting Presymptomatic Infection Is Necessary to Forecast Major Epidemics in the Earliest Stages of Infectious Disease Outbreaks
Source: PLoS Comput Biol. 2016 Apr 5;12(4):e1004836. doi: 10.1371/journal.pcbi.1004836 (PMC4821482; doi:10.1371/journal.pcbi.1004836)

S5 Fig. Robustness of results to different ratios of incubation to infectious period. For extremely short incubation periods, the probability of a major outbreak can be estimated more accurately (since variation in the number of presymptomatic infected individuals between simulations is lower). Here, the infectious period is held fixed and the incubation period varied so that the ratio of these is consistent with poliomyelitis (ratio = 0.12), influenza (ratio = 0.8), mumps (ratio = 2.5) and diphtheria (ratio = 5) [4]. True probabilities greater than 0.97 are classified into bins of size 0.01.

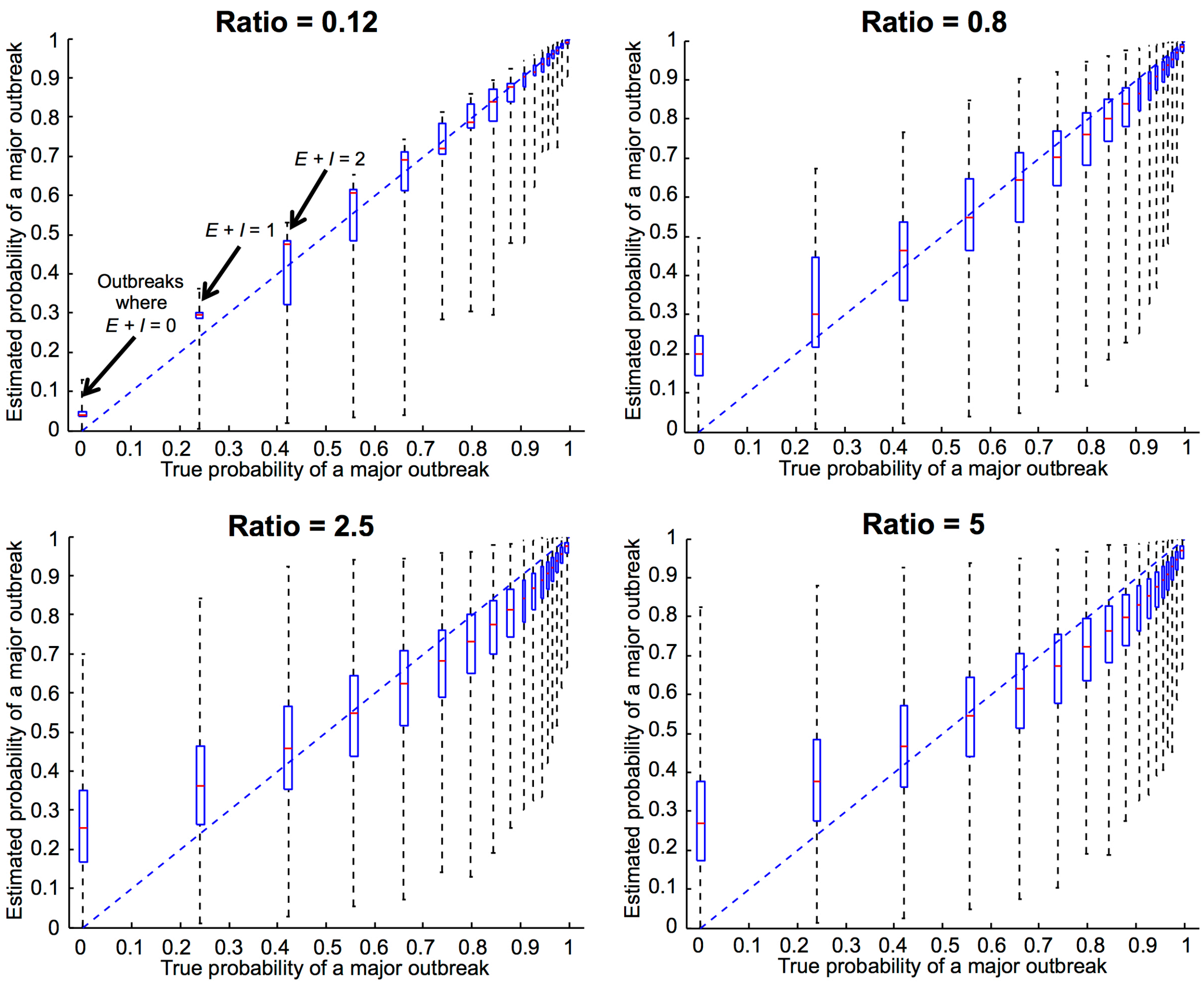

Supplement: S5 Fig — For extremely short incubation periods, the probability of a major outbreak can be estimated more accurately (since variation in the number of presymptomatic infected individuals between simulations is lower). Here, the infectious period is held fixed and the incubation period varied so that the ratio of these is consistent with poliomyelitis (ratio = 0.12), influenza (ratio = 0.8), mumps (ratio = 2.5) and diphtheria (ratio = 5) [4]. True probabilities greater than 0.97 are classified into bins of size 0.01. (PDF) [file pcbi.1004836.s005.pdf]
