# Supplementary material for: Detecting Presymptomatic Infection Is Necessary to Forecast Major Epidemics in the Earliest Stages of Infectious Disease Outbreaks
Source: PLoS Comput Biol. 2016 Apr 5;12(4):e1004836. doi: 10.1371/journal.pcbi.1004836 (PMC4821482; doi:10.1371/journal.pcbi.1004836)

S6 Fig. Robustness of results to different values of the basic reproductive number. R_0 is varied by changing the infection rate, β, between subfigures. For R_0 = 1.2, true probabilities greater than 0.97 are classified into bins of size 0.01. For R_0 = 1.6, true probabilities greater than 0.98 are classified into bins of size 0.01. For R_0 = 2 and R_0 = 4, true probabilities greater than 0.99 are classified into a bin of size 0.01.

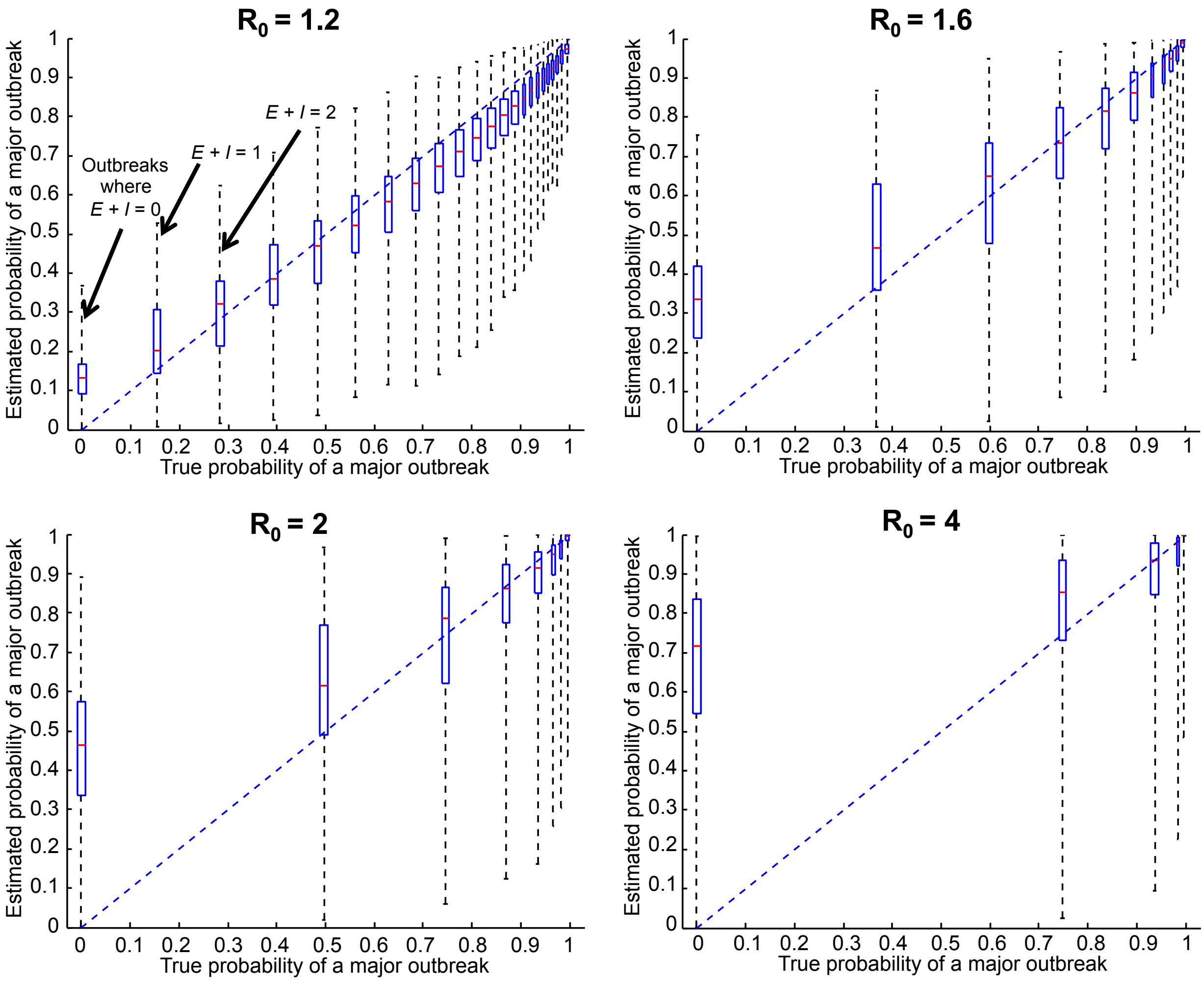

Supplement: S6 Fig — R0 is varied by changing the infection rate, β, between subfigures. For R0 = 1.2, true probabilities greater than 0.97 are classified into bins of size 0.01. For R0 = 1.6, true probabilities greater than 0.98 are classified into bins of size 0.01. For R0 = 2 and R0 = 4, true probabilities greater than 0.99 are classified into a bin of size 0.01. (PDF) [file pcbi.1004836.s006.pdf]
