# Supplementary material for: Detecting Presymptomatic Infection Is Necessary to Forecast Major Epidemics in the Earliest Stages of Infectious Disease Outbreaks
Source: PLoS Comput Biol. 2016 Apr 5;12(4):e1004836. doi: 10.1371/journal.pcbi.1004836 (PMC4821482; doi:10.1371/journal.pcbi.1004836)

S7 Fig. Error in the probability of a major outbreak when the infection rate is estimated. If the probability of a major outbreak is estimated with β unknown but the number of presymptomatic infected individuals at the time of estimation known, then there can be less error than due to presymptomatic infection alone. Here, β is estimated via maximum likelihood estimation, and constrained to within x% of the true value (representing prior knowledge about the value of β). Constructed from 10,000 outbreaks simulated until four deaths have occurred using the SEIR model.

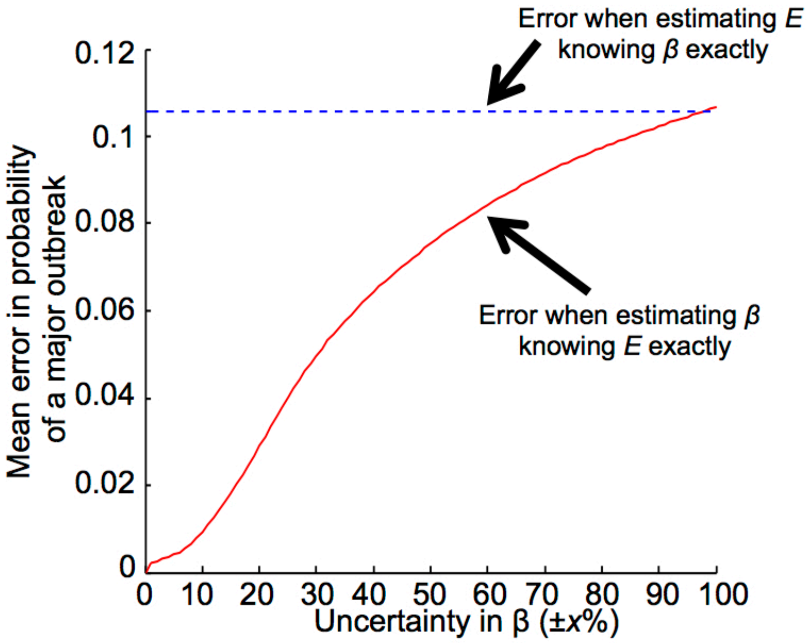

Supplement: S7 Fig — If the probability of a major outbreak is estimated with β unknown but the number of presymptomatic infected individuals at the time of estimation known, then there can be less error than due to presymptomatic infection alone. Here, β is estimated via maximum likelihood estimation, and constrained to within x% of the true value (representing prior knowledge about the value of β). Constructed from 10,000 outbreaks simulated until four deaths have occurred using the SEIR model. (PDF) [file pcbi.1004836.s007.pdf]
