# Supplementary material for: Detecting Presymptomatic Infection Is Necessary to Forecast Major Epidemics in the Earliest Stages of Infectious Disease Outbreaks
Source: PLoS Comput Biol. 2016 Apr 5;12(4):e1004836. doi: 10.1371/journal.pcbi.1004836 (PMC4821482; doi:10.1371/journal.pcbi.1004836)

S8 Fig. Error in estimating the probability of a major outbreak when asymptomatic individuals are tested for infection, when the incubation and latent periods are not identical. The mean error in the probability of a major outbreak, as a function of the percentage of asymptomatic individuals tested and the probability that presymptomatic infection is correctly identified, is calculated from 10,000 simulations for each (percentage, p_d) pair when: A. Symptoms appear before individuals become infectious; B. Symptoms appear after individuals become infectious.

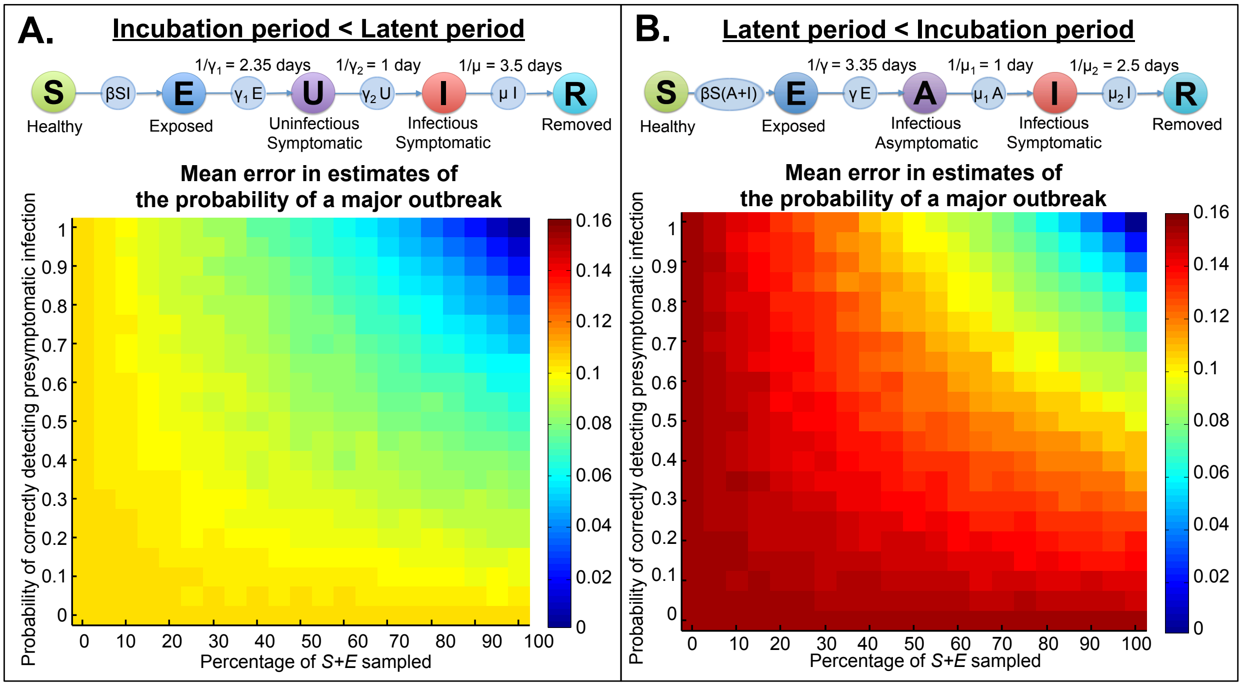

Supplement: S8 Fig — The mean error in the probability of a major outbreak, as a function of the percentage of asymptomatic individuals tested and the probability that presymptomatic infection is correctly identified, is calculated from 10,000 simulations for each (percentage, pd) pair when: A. Symptoms appear before individuals become infectious; B. Symptoms appear after individuals become infectious. (PDF) [file pcbi.1004836.s008.pdf]
