# Supplementary material for: Detecting Presymptomatic Infection Is Necessary to Forecast Major Epidemics in the Earliest Stages of Infectious Disease Outbreaks
Source: PLoS Comput Biol. 2016 Apr 5;12(4):e1004836. doi: 10.1371/journal.pcbi.1004836 (PMC4821482; doi:10.1371/journal.pcbi.1004836)

S9 Fig. Estimating the probability of a major outbreak with gamma distributed incubation and infectious periods. The incubation and infectious periods are both split into three classes, each with exponentially distributed waiting times. Since the true probability of a major outbreak is no longer restricted to discrete values, we classify the true probabilities into bins. The bins are of size 0.1 for true probabilities greater than 0.3. Since lower true probabilities occur infrequently, for computational efficiency we then consider true probabilities equal to 0, and true probabilities greater than zero but less than 0.3, in their own bins.

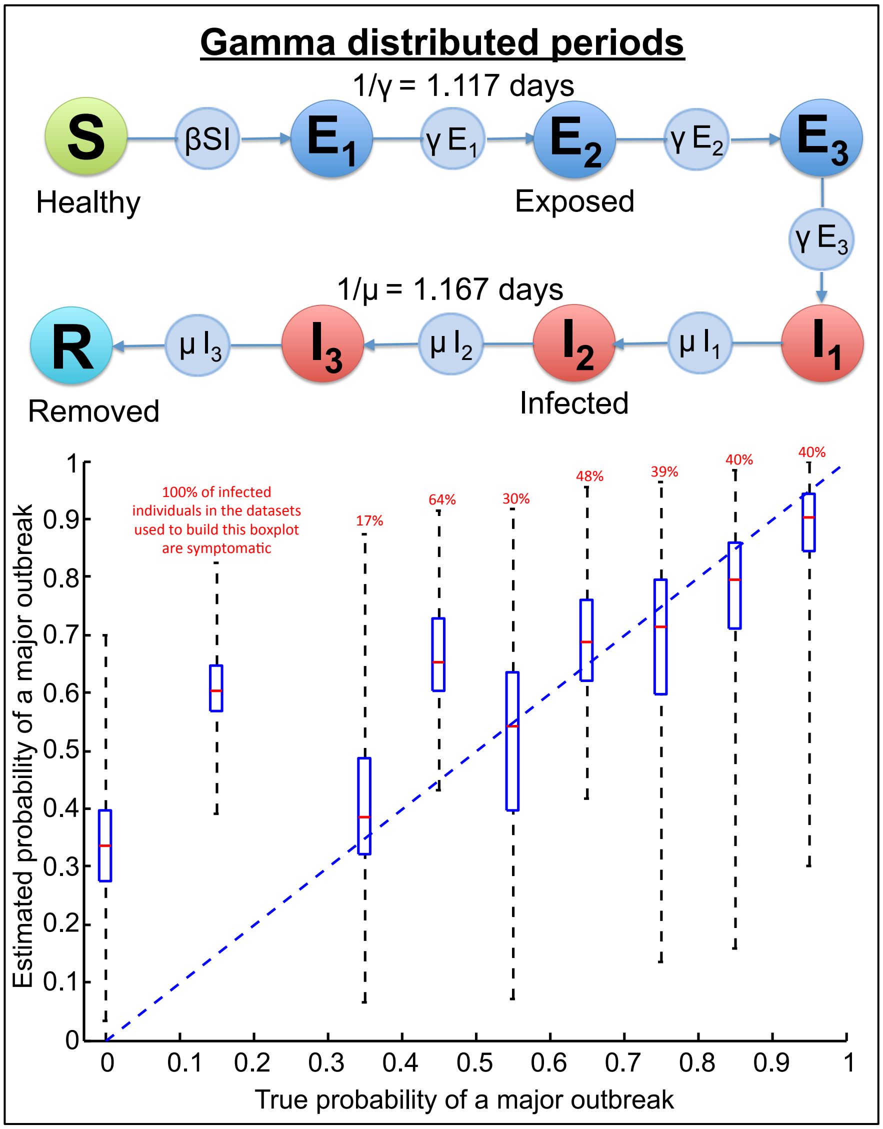

Supplement: S9 Fig — The incubation and infectious periods are both split into three classes, each with exponentially distributed waiting times. Since the true probability of a major outbreak is no longer restricted to discrete values, we classify the true probabilities into bins. The bins are of size 0.1 for true probabilities greater than 0.3. Since lower true probabilities occur infrequently, for computational efficiency we then consider true probabilities equal to 0, and true probabilities greater than zero but less than 0.3, in their own bins. (PDF) [file pcbi.1004836.s009.pdf]
