# Supplementary material for: Detecting Presymptomatic Infection Is Necessary to Forecast Major Epidemics in the Earliest Stages of Infectious Disease Outbreaks
Source: PLoS Comput Biol. 2016 Apr 5;12(4):e1004836. doi: 10.1371/journal.pcbi.1004836 (PMC4821482; doi:10.1371/journal.pcbi.1004836)

S10 Fig. Using a single simulated dataset to compare the exact probability distribution for the current epidemic size given idealized data, as used in our paper, with approximations of this distribution obtained using back-calculation (see Text S4). A. The dataset used for estimating *E* (the true value of *E* at the time of estimation is 2). B. Estimation of *E* using simple back-calculation. As can be seen, simple back-calculation does not discriminate well between different small values of *E*, which is important in estimating the probability of a major outbreak when the first few symptomatic cases occur. C. Estimation of *E* using extended back-calculation (with the full *I(t)* curve observed, and population size and transmission parameters known). Extended back-calculation captures the exact distribution used in our manuscript.

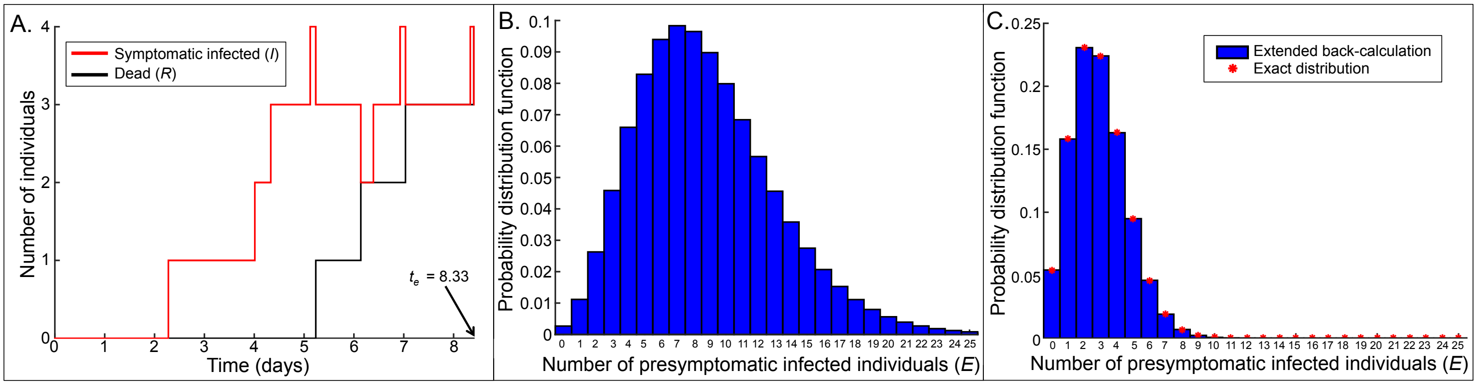

Supplement: S10 Fig — A. The dataset used for estimating E (the true value of E at the time of estimation is 2). B. Estimation of E using simple back-calculation. As can be seen, simple back-calculation does not discriminate well between different small values of E, which is important in estimating the probability of a major outbreak when the first few symptomatic cases occur. C. Estimation of E using extended back-calculation (with the full I(t) curve observed, and population size and transmission parameters known). Extended back-calculation captures the exact distribution used in our manuscript. (PDF) [file pcbi.1004836.s010.pdf]
