# Supplementary material for: Detecting Presymptomatic Infection Is Necessary to Forecast Major Epidemics in the Earliest Stages of Infectious Disease Outbreaks
Source: PLoS Comput Biol. 2016 Apr 5;12(4):e1004836. doi: 10.1371/journal.pcbi.1004836 (PMC4821482; doi:10.1371/journal.pcbi.1004836)
Supplement: S1 Text — (PDF) [file pcbi.1004836.s011.pdf]

S1 Text. Estimating the number of presymptomatic infected individuals

So that the probability of a major outbreak can be estimated, it is necessary to first estimate the current size of the outbreak. The method used to estimate the number of presymptomatic infected individuals necessarily depends on the model in question.

(1) Equal incubation and latent periods (SEIR model)

We use the dynamics of the outbreak up to the time of estimation, t_e, to build a probability distribution function for the estimated value of E. This is a binomial distribution, where the probability that each asymptomatic individual is presymptomatically infected is

$$\text{Prob(asymptomatic individual is infected)} = \frac{\mathrm{Prob}(E)}{\mathrm{Prob}(S)+\mathrm{Prob}(E)}, \qquad \text{(S1)}$$

where

$$\mathrm{Prob}(S) = \exp\left(-\beta\int_0^{t_e} I(s)\,\mathrm{d}s\right),$$

namely the probability that the host remains uninfected until time t_e, and

$$\mathrm{Prob}(E) = \int_0^{t_e} \beta I(t)\exp\left(-\beta\int_0^{t} I(s)\,\mathrm{d}s\right)\times\exp\left(-\gamma(t_e-t)\right)\mathrm{d}t\,.$$

The first part of the Prob(E) integrand represents the probability of the host becoming infected at time t, and the second that the host does not then become symptomatic by time t_e. We assume that the E to I transition times, the I to R transition times (matched, where possible, to an E to I transition time), the current numbers of I and R, and the start time of the epidemic, are all known, so that any error in estimates is due to presymptomatic infection alone.

(2) Unequal incubation and latent periods (SEUIR and SEAIR models)

In the case of the SEUIR model, for which the incubation period is shorter than the latent period, the same calculation can be carried out. In this case, we assume that the exact distribution of individuals between the U and I classes is known: whilst this is impossible in practice for real data, this allows us to quantify the error in the probability of a major outbreak due to presymptomatic infection alone. We note that this distribution could itself be estimated using the time since symptom onset of each individual.

If the incubation period is longer than the latent period (the SEAIR model case), a calculation analogous to equation (S1) is impossible because the infectious pressure on susceptible individuals throughout the epidemic is unknown due to asymptomatic infectious individuals. Instead, reversible jump Markov chain Monte Carlo [1] (RJMCMC) is used to estimate whether asymptomatic individuals are in the S, E or A classes. Probability distributions for S, E and A are

obtained by sampling from the RJMCMC output. For clarity, we separate out the description of the RJMCMC algorithm from this section, and describe it in Text S2.

(3) Gamma-distributed incubation and infectious periods

When gamma distributions are used for the exposed and infectious periods, the probability that an asymptomatic individual is infected is calculated according to equation (S1). The second term of the integrand of Prob(E) is replaced by the cumulative distribution function of a gamma distributed random variable. For the purpose of forward simulations, we note that the probability of a major outbreak is independent of how the exposed individuals are distributed between the exposed classes, since these individuals are all likely to spend the same period of time infectious in future. In calculations of both the true and estimated probability of a major outbreak, each infectious individual is placed in an I compartment chosen as follows: the time since symptom onset is used to construct the probability that the individual is in each of the I compartments, and the compartment for each individual simulation is chosen randomly according to these probabilities. This is equivalent to, in each forward simulation, sampling the infectious periods of currently infectious individuals from gamma distributions conditioned on still being infectious at the time of estimation, i.e. an exactly analogous method to that used for the simple SEIR model.

Reference

1. Green PJ. Reversible jump Markov chain Monte Carlo computation and Bayesian model determination. Biometrika. 1995;82: 711–732.
