# Supplementary material for: Detecting Presymptomatic Infection Is Necessary to Forecast Major Epidemics in the Earliest Stages of Infectious Disease Outbreaks
Source: PLoS Comput Biol. 2016 Apr 5;12(4):e1004836. doi: 10.1371/journal.pcbi.1004836 (PMC4821482; doi:10.1371/journal.pcbi.1004836)
Supplement: S2 Text — (PDF) [file pcbi.1004836.s012.pdf]

S2 Text. Estimating the number of individuals in each compartment of the SEAIR model

When the incubation period is longer than the latent period and the SEAIR model is used (Figs. 4B and S8B), reversible jump Markov chain Monte Carlo [1] (RJMCMC) is used to estimate whether asymptomatic individuals are in the S, E or A classes. We describe this process here.

1. Each asymptomatic individual is assigned at random an initial guess as to whether they are in the S, E or A class. Randomly chosen times of transition into the E and A classes are assigned to individuals in those classes. For individuals known to be in the I or R classes, randomly chosen times of transition from S to E and E to A are proposed.

2. The following steps will be repeated M times. New transition times are proposed, with one of the following proposals chosen uniformly at random:

   - Case 1: An S to E time is moved,
   - Case 2: An E to A time is moved,
   - Case 3: An individual in the S class is assigned infection times,
   - Case 4: An infection is removed.

   In case 3, one of two proposals is made (each with probability 0.5). Either, the susceptible individual has only an S to E time proposed, or both S to E and E to A times are proposed. In the second of these subcases, the time to propose first is chosen uniformly at random. In case 4, one of two proposals is made (each with probability 0.5). Either an individual in the E class (according to the current state of the RJMCMC chain) has their infection time removed, or an individual in the A class has both their S to E and E to A times removed. If a transition time is added, it is chosen uniformly at random in the interval of possible times: for example, if an E to A time is added for an individual known never to become I, and whose S to E time has already been assigned, the new time is proposed uniformly at random on the interval [S to E time, t_e].

3. Denote the likelihood of the proposed times given the data by $L(2)$, and the likelihood of the previous set of times given the data by $L(1)$. Accept the proposed transition times with probability:

   - Cases 1 & 2: $\min\left(\frac{L(2)}{L(1)},1\right)$
   - Cases 3 & 4: $\min\left(\frac{L(2)}{L(1)}\frac{p_r}{p_p},1\right)$

where t_e is the time of estimation. The expression p_p is the probability that the move concerned was chosen, and p_r the probability that, if the proposed move is accepted, this move is reversed at the next step through this algorithm. Direct comparison of likelihoods $L(2)$ and $L(1)$ is incorrect if there are different numbers of current and proposed transition times, and the factor p_r/p_p accounts for this. For example, if case 3 occurs and an individual in the S class is assigned an S to E transition time, then

$$p_p = \frac{1}{4} \times \frac{1}{2} \times \frac{1}{n_S} \times \frac{1}{t_e},$$

i.e. the product of the probability of case 3 happening, the probability that just an S to E time is added, the probability that the individual to add the infection time to is the individual chosen (i.e. there are n_S individuals in the S class before the proposal), and the probability density of the proposed time being the time chosen (since times are chosen uniformly on $[0, t_e]$). Denoting the S to E time of individual i by e_i, with similar notation for other transition times, the likelihood is given by

$$L = L_1 \times L_2,$$

where L_1 is the infections component of the likelihood, and L_2 corresponds to the behavior of individuals post infection. The first of these is of the form

$$L_1 = \prod_{j \in v_E} \left(\beta(A(e_j) + I(e_j))\right) \exp\left(-\beta \int_0^{t_e} (A + I) S \,\mathrm{d}t\right),$$

where v_E is the set of individuals who ever become infected (excluding the initial infected). The L_2 term is of the form

$$\begin{aligned} L_2 = & \prod_{j \in M_E} \exp(-\gamma(t_e - e_j)) \times \prod_{j \in M_A} \gamma \exp(-\gamma(a_j - e_j)) \exp(-\mu_1(t_e - a_j)) \times \\ & \prod_{j \in M_I} \gamma \exp(-\gamma(a_j - e_j)) \mu_1 \exp(-\mu_1(i_j - a_j)) \exp(-\mu_2(t_e - i_j)) \times \\ & \prod_{j \in M_R} \gamma \exp(-\gamma(a_j - e_j)) \mu_1 \exp(-\mu_1(i_j - a_j)) \mu_2 \exp(-\mu_2(r_j - i_j)), \end{aligned}$$

where M_E is the set of individuals in the E class at time t_e (with similar meanings for M_A, M_I and M_R).

4. Repeat this procedure from step 2 until M jumps have occurred.

The RJMCMC algorithm is run for M = 100,000 jumps, and the first 50,000 jumps are discarded as a burn-in period for the algorithm to explore parameter space. The second 50,000 jumps are used to construct a joint probability distribution for S, E and A. Initial conditions for forward simulations are sampled from this distribution, to estimate the probability of a future major outbreak. The

convergence of the RJMCMC algorithm with this burn-in and number of jumps was assessed by eye, and the results were also confirmed to match Fig. 2 when the proportion of time infectious individuals spend in the *A* class was set to zero.

Reference

1. Green PJ. Reversible jump Markov chain Monte Carlo computation and Bayesian model determination. Biometrika. 1995;82: 711–732.
