# Supplementary material for: Detecting Presymptomatic Infection Is Necessary to Forecast Major Epidemics in the Earliest Stages of Infectious Disease Outbreaks
Source: PLoS Comput Biol. 2016 Apr 5;12(4):e1004836. doi: 10.1371/journal.pcbi.1004836 (PMC4821482; doi:10.1371/journal.pcbi.1004836)
Supplement: S3 Text — (PDF) [file pcbi.1004836.s013.pdf]

S3 Text. Improving estimates by sampling to find presymptomatic infection

As described in the Methods, testing asymptomatic individuals to find presymptomatic infection can make the probability distribution for the current size of the outbreak more precise. The probability of correctly identifying presymptomatic infection when an infected individual is tested is denoted by p_d. If E_n is the number of presymptomatic infected individuals correctly identified as infected out of a sample of n asymptomatic individuals, and there are m asymptomatic individuals (including susceptibles) in total, then

$$\mathrm{P}(E = e \mid E_n = k) = \frac{\mathrm{P}(E_n = k \mid E = e)\mathrm{P}(E = e)}{\sum_{j=k}^{m} \mathrm{P}(E_n = k \mid E = j)\mathrm{P}(E = j)},$$

valid for $e = k, k+1, \ldots, m$, where the final term of the numerator, and the final term of the denominator, can be calculated described in the previous section (i.e. according to equation (S1) in Text S1, or in the case where the incubation period is longer than the latent period, from the posterior from RJMCMC). The first terms of the numerator and denominator are given by

$$\mathrm{P}(E_n = k \mid E = j) = \begin{cases} \dfrac{\binom{j}{k}\binom{m-j}{n-k}}{\binom{m}{n}} & \text{for } p_d = 1, \\[2ex] \displaystyle\sum_{l=\max(0,n-k-m+j)}^{\min(j,n)-k} \frac{\binom{j}{k+l}\binom{m-j}{n-k-l}\binom{k+l}{k} p_d^{\,k}(1-p_d)^l}{\binom{m}{n}} & \text{for } p_d \in [0,1), \end{cases}$$

where the second expression is valid whenever $\min(j, n) - k \geq \max(0, n-k-m+j)$, and is zero otherwise.
