# Supplementary material for: Detecting Presymptomatic Infection Is Necessary to Forecast Major Epidemics in the Earliest Stages of Infectious Disease Outbreaks
Source: PLoS Comput Biol. 2016 Apr 5;12(4):e1004836. doi: 10.1371/journal.pcbi.1004836 (PMC4821482; doi:10.1371/journal.pcbi.1004836)
Supplement: S4 Text — (PDF) [file pcbi.1004836.s014.pdf]

S4 Text. Consistency of our estimates with an extended version of back-calculation

Back-calculation, as was originally introduced by Brookmeyer and Gail to estimate the size of the HIV-AIDS epidemic in the 1980s [1,2], combines reports of symptomatic cases with information on the distribution of incubation periods to estimate the total number of individuals currently infected during an established epidemic. The advantages of the method are that it is purely statistical (i.e. does not require any simulation of outbreaks), and can be used even when there are several unknown variables in the system (such as the total population size, and the values of disease transmission parameters). Back-calculation is widely used [3-8,9], and provides a methodology for estimating the number of presymptomatic infected individuals, which is an input for the simulation methods presented in our paper.

To test whether major outbreaks can ever be predicted from symptomatic case data alone, we used the exact distribution for the number of infected individuals in the light of presymptomatic infection. If this distribution has to be estimated, for example via back-calculation, estimates of the number of presymptomatic infected individuals are necessarily less precise. Consequently, the calculations we present in the main text of the paper are an upper bound on the accuracy of forecasts using symptomatic cases data alone. We illustrate this below. However, we also go on to show how the naïve estimate obtained from simple back-calculation methods can be improved with complete data on symptomatic cases, and knowledge of the values of disease transmission parameters and the total population size, that we assume are available. This improved version of back-calculation leads to an identical distribution to that used in our paper, showing that our method and back-calculation are consistent.

Estimating the number of presymptomatic infecteds for the SEIR model

In the SEIR model case, each of the N individuals in our population is in one of four epidemiological classes: (S)usceptible, (E)xposed, (I)nfected or (R)emoved. Exposed individuals are presymptomatic, i.e. are infected but do not yet show symptoms. We estimate a probability distribution for the number of exposed individuals using data from the symptomatic (I) cases. We perform estimation at time t_e, by which time m individuals have become infected or removed (i.e. $I + R = m$).

Since we assume we know the full time course of the number of symptomatic infected individuals, $I(t)$, we can write down probabilities that a randomly-chosen individual is in any of the S, E, I or R classes. In particular

$$p_S = \exp\left(-\beta \int_0^{t_e} I(s)\,\mathrm{d}s\right),$$

$$p_E = \int_0^{t_e} \beta I(t) \exp\left(-\beta \int_0^{t} I(s)\,\mathrm{d}s\right) \times \exp(-\gamma(t_e - t))\,\mathrm{d}t,$$

$$p_I = \int_0^{t_e} \int_t^{t_e} \beta I(t) \exp\left(-\beta \int_0^{t} I(s)\,\mathrm{d}s\right) \times \gamma \exp(-\gamma(u - t)) \times \exp(-\mu(t_e - u))\,\mathrm{d}u\mathrm{d}t,$$

$$p_R = \int_0^{t_e}\int_t^{t_e}\int_u^{t_e} \beta I(t)\exp\left(-\beta\int_0^t I(s)\,\mathrm{d}s\right)\times\gamma\exp(-\gamma(u-t))\times\mu\exp(-\mu(v-u))\,\mathrm{d}v\mathrm{d}u\mathrm{d}t.$$

The probability that a randomly-chosen asymptomatic individual is infected is then simply

$$p = \mathrm{P}(E \mid S \text{ or } E) = \frac{p_E}{p_S + p_E},$$

cf. Eqn. (S1) in Text S1. The number infected of the $N - m$ individuals that are not showing symptoms is then binomially distributed, with

$$\mathrm{P}(E = e \mid I + R = m) = \binom{N-m}{e} p^{N-m-e}(1-p)^e. \qquad \text{(S2)}$$

We use this closed-form expression for the distribution of the number of presymptomatic infected individuals in the simulations we present.

<u>Standard back-calculation</u>

Back-calculation instead estimates the probability that a randomly chosen infected individual is symptomatic. Typically this probability is estimated conjointly with the number infected from the case report data. However, since we have full data on all epidemiological transitions of symptomatic individuals, we can in fact simply write down this probability

$$q = \mathrm{P}(I \text{ or } R | E \text{ or } I \text{ or } R) = \frac{p_I + p_R}{p_E + p_I + p_R}.$$

This allows us to write a likelihood function that there are n infected individuals, namely the probability of observing the number of symptomatic infections actually seen given there are $E+I+R = n$ infected individuals

$$L(n) = \mathrm{P}(I + R = m | E + I + R = n) = \binom{n}{m} q^m (1-q)^{n-m}.$$

Back-calculation then leads to a point estimate of the number of presymptomatic infections by maximizing this likelihood. If desired, confidence intervals can also be estimated via standard techniques, namely by inverting a likelihood ratio test.

<u>Extending back-calculation</u>

What we actually require to drive our forward simulations is the full distribution of the number of presymptomatic infected individuals. Assuming the relevant probabilities could be calculated, this would immediately follow from the above likelihood function via Bayes' theorem

$$\mathrm{P}(E = n - m \mid I + R = m) = \frac{L(n)\mathrm{P}(E+I+R=n)}{\mathrm{P}(I+R=m)}.$$

The terms on the right-hand side of this expression typically cannot be estimated. However, we can use our epidemic model, our rich case data and our knowledge of the transmission parameter values and total population size to calculate the requisite probabilities. In particular

$$\mathrm{P}(E = n - m \mid I + R = m) = \frac{\binom{n}{m} q^m (1-q)^{n-m} \binom{N}{n} (p_E + p_I + p_R)^n (p_S)^{N-n}}{\binom{N}{m} (p_I + p_R)^m (p_S + p_E)^{N-m}}.$$

Following some algebra, this reduces to exactly the expression in equation (S2) above, with $e = n - m$. Consequently, this extended version of back-calculation leads to exactly the same distribution for E that we use in the paper.

Numerical example

To illustrate the methodology, we consider a dataset created using the stochastic SEIR model under the same conditions as in the main text of the paper, i.e. until the fourth death occurs. We apply the estimation method used in the main text of the paper to give the true distribution of the number of presymptomatic infected individuals given presymptomatic infection. We then also apply the simple version of back-calculation and our extended version of back-calculation to this dataset to obtain distributions for the number of presymptomatic infected individuals. The dataset is shown in Fig. S10A, and in Fig. S10B is the likelihood distribution for E obtained using simple back-calculation. Fig. S10C shows the extended version of back-calculation (blue) with the true distribution obtained as in the main text of the paper (red stars). As can be seen, the true distribution of the number of presymptomatic infected individuals is significantly more precise than the distribution obtained using simple back-calculation, but the exact distribution is obtained when back-calculation is extended in the light of perfect knowledge of symptomatic cases, total population size and parameter values.

References

1. Brookmeyer R, Gail MH. Minimum size of the acquired immunodeficiency syndrome (AIDS) epidemic in the United States. Lancet. 1986;328: 1320-1322.

2. Brookmeyer R, Gail MH. A method for obtaining short-term projections and lower bounds on the size of the AIDS epidemic. J Am Statist Ass. 1988;83: 301-308.

3. Brookmeyer R. Reconstruction and future trends of the AIDS epidemic in the United States. Science. 1991;253: 37-42.

4. Rosenberg PS, Gail MH. Backcalculation of flexible linear models of the human immunodeficiency virus infection curve. Appl Stat. 1991;2: 269-282.

5. Biggar RJ, Rosenberg PS. HIV infection/AIDS in the United States during the 1990s. Clin Inf Dis. 1993;17: S219-223.

6. Bacchetti P, Segal MR, Jewell NP. Backcalculation of HIV infection rates. Stat Sci. 1993;8: 82-101.

7. Jewell NP, Lu BW. Some variants of the backcalculation method for estimation of disease incidence: an application to multiple sclerosis data from the Faroe Islands. Int J Biostat. 2005;1: 1-24.

8. Hall HI, Song R, Rhodes P, Prejean J, An Q, Lee LM *et al*. Estimation of HIV incidence in the United States. J Am Med Assoc. 2008;300: 520-529.

9. Egan JR, Hall IM. A review of back-calculation techniques and their potential to inform mitigation strategies with application to non-transmissible acute infectious diseases. J R Soc Interface. 2015;12: 20150096.
